# Development and clinical validation of a 3-miRNA signature to predict prognosis of gastric cancer

**DOI:** 10.7717/peerj.10462

**Published:** 2021-02-03

**Authors:** Wenqian Qi, Qian Zhang

**Affiliations:** Department of Gastroenterology, China-Japan Union Hospital, Jilin University, Changchun, Jilin Province, China

**Keywords:** Gastric cancer, miRNA signature, GEO, Prognosis

## Abstract

**Aims:**

Identification of miRNA signature to predict the prognosis of gastric cancer (GC) patients by integrating bioinformatics and experimental validation.

**Methods:**

The miRNA expression profile and clinical data of GC were collected. The univariable and LASSO-Cox regression were used to construct the risk signature. The receiver operating characteristic (ROC) curve analysis confirmed the good performance of the prognostic model.

**Results:**

A 3-miRNA prognostic signature was constructed, which included hsa-miR-126-3p, hsa-miR-143-5p, and hsa-miR-1275. A nomogram, including the prognostic signature to predict the overall survival, was established, and internal validation in the The Cancer Genome Atlas (TCGA) cohort was performed. We found that compared with the traditional pathological stage, the nomogram was the best at predicting the prognosis.

**Conclusions:**

The predictive model and the nomogram will enable patients with GC to be more accurately managed in clinical practice.

## Introduction

Gastric cancer (GC) is one of the most common malignant tumors globally, which seriously threatens the health and life expectancy of those affected. According to the latest statistics from GLOBOCAN, there were 1.033 million new cases of GC worldwide and approximately 783,000 deaths from the disease in 2008 (Ferlay et al., 2019). Surgical resection can be effective in the early treatment of GC (Sun et al., 2011). However, patients with gastric cancer often lack typical symptoms at an early stage. As a result, over 70% of patients are already in the advanced stage of gastric cancer at the time of diagnosis, so gastric cancer often has a poor prognosis (Song et al., 2017). High rates of invasiveness and metastasis are also important causes of poor prognosis in GC (Mayer, Venook & Schilsky, 2014; Deng & Liang, 2014).

MiRNA regulates the expression of a variety of cancer-related genes in cells while participating in the cell cycle, apoptosis, proliferation, differentiation, and other processes (Friedman et al., 2009; Wang et al., 2017; Shi et al., 2016; Zheng et al., 2017; Lima et al., 2016; Stevanato et al., 2016; Momen-Heravi et al., 2015). The occurrence of tumors is often accompanied by changes in the expression of some specific miRNA. There is obviously tissue specificity, and there are different mechanisms in different types of tumors and different pathological stages. Moreover, miRNA can exist stably in the blood and other body fluids. This feature makes it possible for miRNA to become a convenient, non-invasive, and effective tumor marker.

MiRNA is involved in the occurrence, metastasis, and prognosis of tumor. There have been multiple studies on miRNA and gastric cancer prognosis. It was revealed that MicroRNA-375 is downregulated in gastric carcinomas and regulates cell survival by targeting PDK1 and 14-3-3zeta (Tsukamoto et al., 2010); miR-218 suppresses gastric cancer cell cycle progression through the CDK6/Cyclin D1/E2F1 axis in a feedback loop (Deng et al., 2017); MiR-196a is upregulated in gastric cancer and promotes cell proliferation by downregulating p27 (Sun et al., 2012); microRNA-451 regulates macrophage migration inhibitory factor production and proliferation of gastrointestinal cancer cells (Bandres et al., 2009). Ueda et al. (2010) discovered that 22 microRNAs in gastric cancer were up-regulated, while 13 genes were down-regulated in 160 non-tumor and cancer paired GC samples, and their histological subtypes were characterized by specific microRNA characteristics.

In the genome era, a large number of genome sequencing techniques and data analyses have made enormous contributions to tumor diagnosis and prognosis prediction (Wang, Gerstein & Snyder, 2009). Gene regulation is a complex network, and researchers began to use sequencing data to build a multi-gene model, combined with multiple gene detection to study tumors. For example, Zhao et al. (2019) used univariate and multivariate Cox regression analysis to identify miRNA related to GC patients’ prognosis from the TCGA-STAD cohort. A predictive risk model based on a 5-miRNA signature was established. Comprehensive analysis of miRNA and mRNA expression profiling chips using univariate and multivariate Cox regression revealed a novel mRNA/miRNA signature that can improve the risk classification of GC patients (Yin et al., 2019). Zhang et al. (2018) used the 20 pairs of GC and normal tissues adjacent to the tumor in the Gene Expression Omnibus (GEO) cohort to identify three prognostic miRNA features (miR-145-3p, miR-125b-5p, and miR-99a-5p).

In this study, we constructed a 3-miRNA signature and nomogram for GC. Comparison with other signatures proves the superiority of our model. Internal validation and experimental verification confirm its accuracy and reliability. It can be used as an independent biomarker to predict GC prognosis, which is conducive to guiding further clinical treatment.

## Materials and Methods

### Data download and preprocessing

The miRNA expression profiles of GC patients were downloaded from GEO using the *GEOquery* package. The profiles were GSE23739 (Oh et al., 2011) and GSE93415 (Sierzega et al., 2017). GSE23739 included 40 gastric cancer tissues and 40 adjacent normal gastric tissue, and GSE93415 included 20 gastric cancer tissues and 20 adjacent normal gastric tissue. At the same time, we used R package *gdcRNAtools* (Li et al., 2018) to download the mature count file of miRNA from the official website of the TCGA database, and the TMM method in the *edgeR* package (Robinson, McCarthy & Smyth, 2010) was used for normalization. In TCGA_STAD, we used the gdcFilterSampleType function in the gdcRNAtools tool (Li et al., 2018) to eliminate non-cancer samples and non-cancer adjacent tissue samples and obtained a total of 477 samples, of which 436 were GC tissues, and 41 were adjacent normal gastric tissue.

### Construction and verification of miRNA risk model

We use the *limma* package (Ritchie et al., 2015) to conduct a difference analysis on GSE23739 and GSE93415 cohort, and set the threshold to |logFC| > 1, *p* < 0.05. We merged the differential miRNAs obtained from GEO cohorts and extracted the expression values of these genes from the TCGA cohort. At the same time, we excluded samples of patients with missing overall survival times. Finally, 409 samples remained in the study. We first used the R package *survival* coxph function (Zhang, 2002) to perform univariable cox analysis to obtain candidate miRNAs. Then, we randomly separated the samples evenly, divided into the training and testing cohorts. We used the “*glmnet*” package (Simon et al., 2011) to perform LASSO-Cox analysis on the selected miRNAs in the training cohort.

LASSO regression is a compressed estimate (Tibshirani, 1996). It constructs a penalty function to obtain a more refined model and sets some coefficients to zero. Therefore, the advantage of subset shrinkage is preserved. It is a kind of processing biased estimation with complex collinearity data, which can perform variable selection at the same time as parameter estimation, and better solve the multicollinearity problem in regression analysis.

Finally, for the selected miRNA, we established a risk score calculation formula, as follows:

\begin{eqnarray*}& & \mathrm{RiskScore}={\mathop{\sum \nolimits }\nolimits }_{i=1}^{N}(\mathrm{exp}\ast \mathrm{coef}) \end{eqnarray*}

in which *N* is the number of genes exp was the expression value of the gene, and coefwas the coefficient of miRNA in the LASSO-Cox regression analysis.

To further evaluate the stability of the model, we used the testing cohort to verify the robustness of the model. We used the same formula to calculate the survival curve. We found that they can also clearly differentiate between prognosis.

### Construction and evaluation of the nomogram model

Nomograms are widely used to predict the prognosis of cancer patients, mainly because they can simplify the statistical prediction model into a single numerical value to assess the survival probability of patients. They use the length of the line to indicate the degree of influence of different variables on the outcome, and the effect of different values of variables on the outcome. The nomogram is applied by adding up the points identified on the points scale for each variable. The total points projected on the bottom scales indicate the probability of 3-y and 5-y’s overall survival.

We used the R package *rms* (Harrell Jr, 2006)to build a nomogram comprising gender, age, TNM stage, lymph node metastases, radiation_therapy, and risk score together.

Calibration plots were used to visualize the performances of the nomograms. The 45° line represented the best prediction. The Decision curve analysis (DCA) is a method for evaluating clinical predictive models, diagnostic tests, and molecular markers. We compared the predictive ability of the nomogram in 3-year and 5-year DCA curves. In order to prove the advantage of the nomogram. We also conducted comparative analyses with other published prognostic models.

### Functional enrichment analysis of miRNA target genes

The target genes co-regulated by three miRNAs were found and intersected by miRTarbase and miRdb database. *ggplot2*.R (Wickham, 2016) package was used to plot bubble chart of Gene Oncology (GO) function and Kyoto Encyclopedia of Genes and Genomes (KEGG)pathway enrichment.

### Real-time quantitative PCR (qPCR) analyses of miRNAs

Twenty pairs of GC and tumor-adjacent normal tissues collected from Department of Gastroenterology, China-Japan Union Hospital, Jilin University were included for validation. For qPCR analyses of miRNAs, qPCR was performed with the stem-loop primers, as reported previously (Raymond et al., 2005). U6 RNA served as an internal control. qPCR was performed with total RNAs, using universal primers and miRNA-specific reverse LNA-primers: miR-126-3p forward: 5′-ACACTCCAGCTGGG TCGTACCGTGAGTAAT- 3′ and reverse: 5′CTCAACTGGTGTCGTGGAGTCGGCAATTCAGTTGAGCGCAT TAT- 3′, miR-1275 forward 5′GTGCAGGGTCCGAGGT-, reverse 5′-GCCGCTAGCTTATCGACTACG- 3′, mir-143-5p forward 5′ATGGTTCGTGGGGTCCAGTTTTCCCAG- 3′ reverse 5′-GTGTCGTGGAGTCGGCAATTC- 3′. U6 RNA served as an internal control, U6 (forward: 5′-CTCG CTTCGGCAGCACA- 3′ and reverse: 5′-A ACGCTTCACGAATTTGCGT- 3′).

### Statistical analysis

The data of miRNA expression in GC and adjacent normal samples were performed by unpaired *t*-test. Kaplan–Meier survival analysis and the univariate/lasso Cox regression analysis were used to identify prognostic miRNA features. All the statistical analyses were performed with R version 3.53 and *P* < 0.05 was considered statistically significant.

## Results

## Construction of miRNA prognosis signature

**Figure 1:**
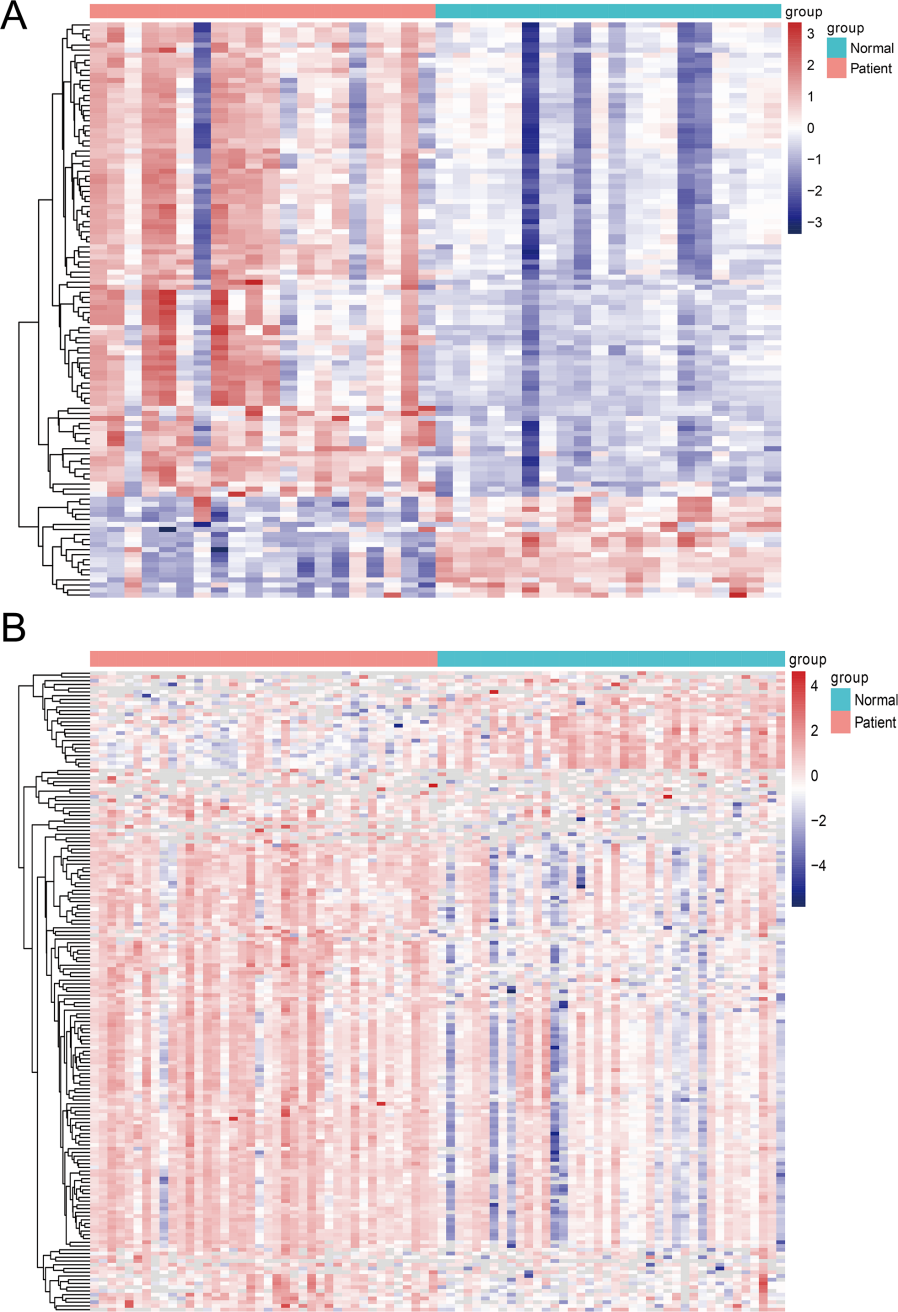
The heatmap of differentially expressed miRNAs between tumor tissues and normal tissues. (A) Differentially expressed miRNAs in the GSE93415 cohort. (B) Differentially expressed miRNAs in the GSE93415 cohort.

A total of 171 differential miRNAs were obtained in the GSE23739 cohort, and a total of 116 differential miRNAs were obtained in the GSE93415 cohort (Tables S1 and S2, Fig. 1). We merged the differentially expressed miRNAs from the GSE23739 and GSE93415 cohorts and obtained 275 miRNAs. Then we extracted the expression values of these differential miRNAs from the TCGA database and identified a total of 138 miRNAs. Univariable Cox analysis resulted in 40 prognostic miRNAs, and the samples from TCGA_STAD cohort were randomly divided into a training set (*N* = 204) and test set (*N* = 205) following 1:1.

**Figure 2:**
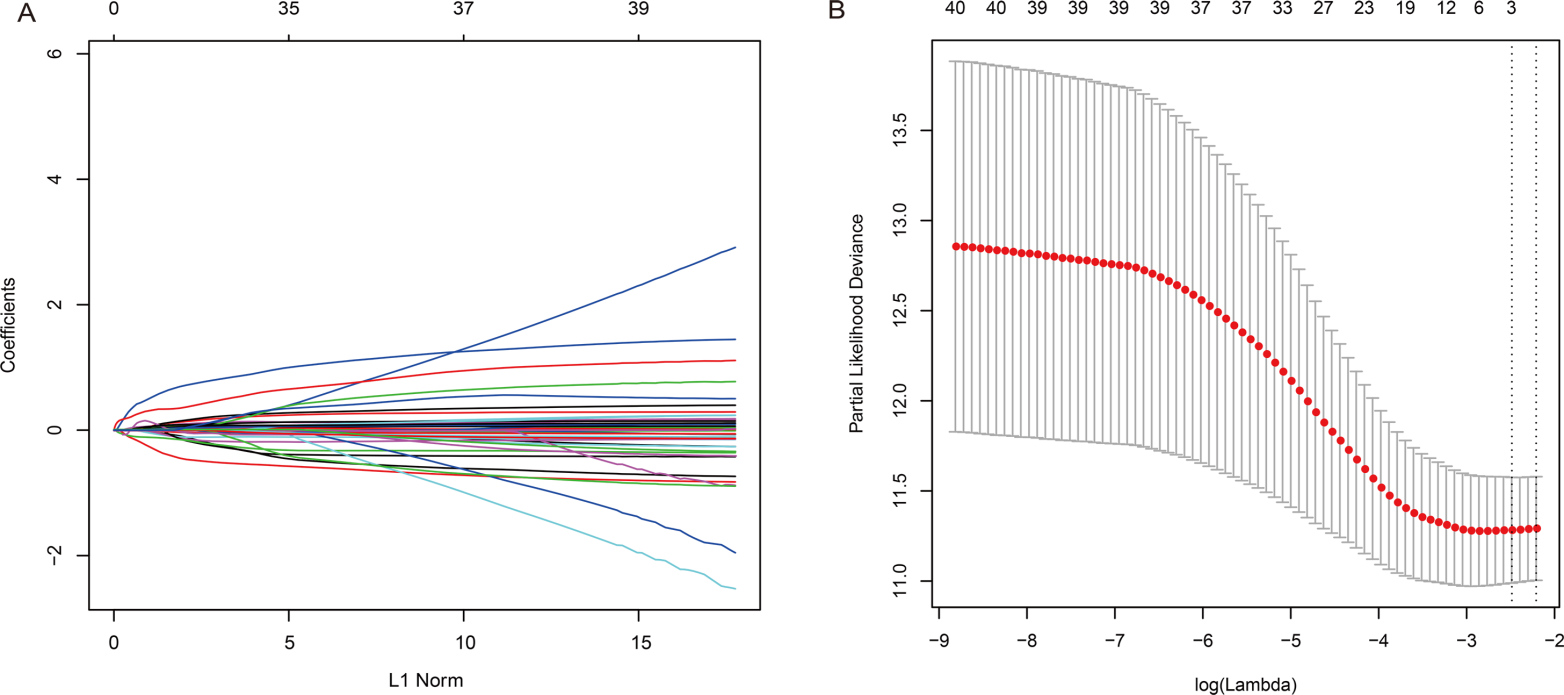
Construction of the 3-miRNA signature model and Selection of optimal tuning parameter in the LASSO model. (A) LASSO coefficient profiles of the 40 associated miRNAs. A vertical line is drawn at the value determined by 10-fold cross validation. (B) The dotted vertical lines are drawn at the optimal values by minimum criteria (left) and 1-standard error (SE) criteria (right). The optimal = 0.0853281 was determined by ten-time cross-validation via minimum criteria. Error bars represent SE.

We analyzed the trajectory of each independent variable by lasso cox analysis (Fig. 2A). It can be observed that as the lambda gradually shrinks, the number of independent variable coefficients tending to 0 gradually increased. We use 10 cross-validation to construct the signature and analyze the confidence interval under each lambda, as shown in Fig. 2B. We can see that the model is optimal when lambda = 0.0853281. At which time, the corresponding number of miRNAs is 3, so, we chose 3 miRNAs (hsa-miR-126-3p, hsa-miR-143-5p, hsa-miR-1275) as the candidate biomarkers.

**Figure 3:**
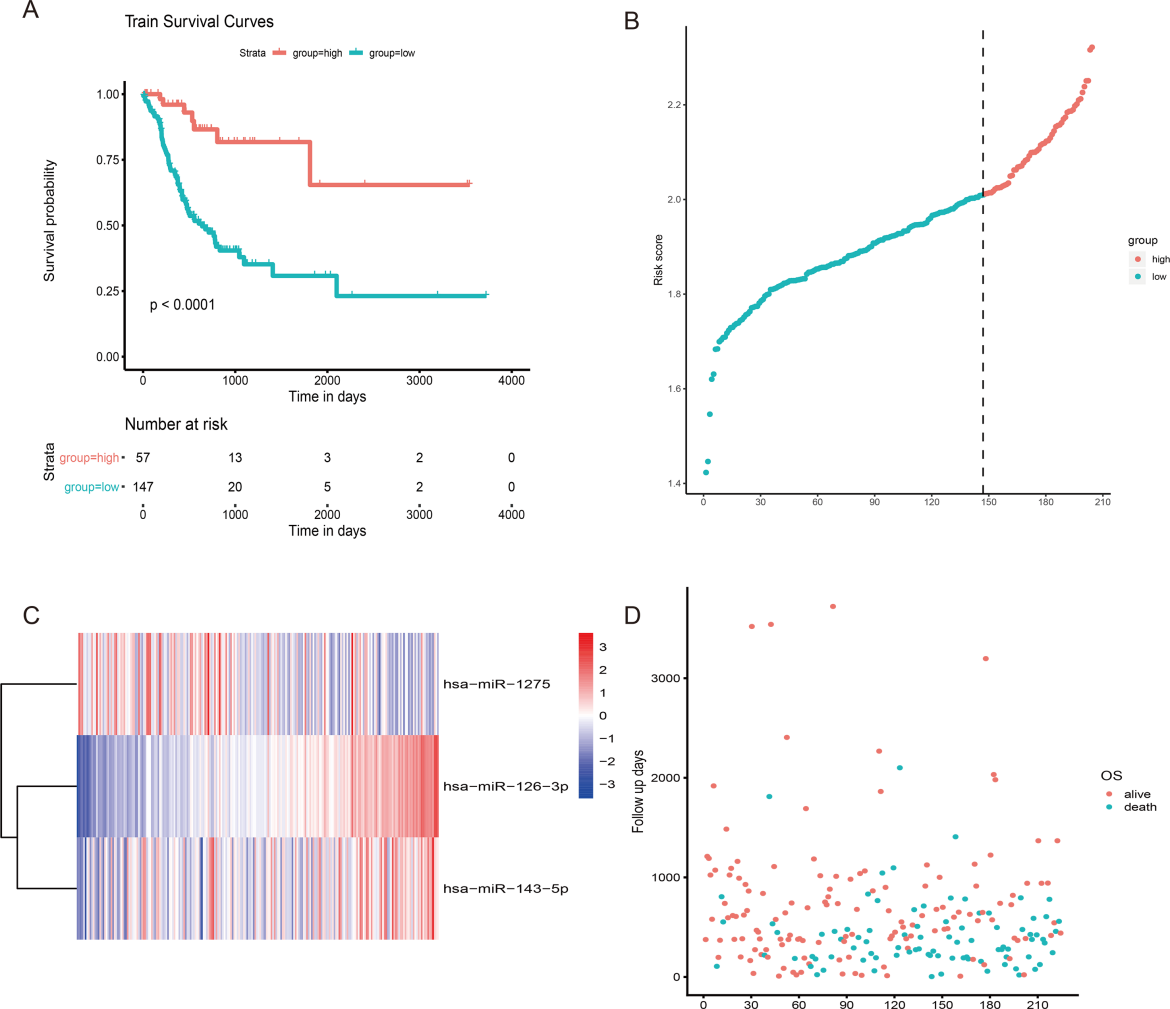
The risk score performance in the training cohort. (A) The survival curve of 3-miRNA riskscore in training cohort.The abscissa represents survival time (days), and the ordinate represents survival rate. The red line represents high expression and the green line represents low expression. (B) The heatmap of gene expression of three miRNA. (C) The risk score distribution of each sample. (D) Distribution of survival time for each sample.

Finally, for the three selected miRNAs, we have established a risk model as follows: Risk score = (0.0829 * expression value of hsa-miR-126-3p) + (0.0191 * expression value of hsa-miR-143-5p) + (−0.0271 * expression value of hsa-miR-1275).

We use the *survmier* package to determine the best cutoff value according to the risk score and classify patients into high-risk (*n* = 159) and low-risk (*n* = 65) groups. Survival analysis showed that patients with high and low risk could be clearly separated (Fig. 3). Then we performed a univariate multivariate cox regression analysis of gender, age, number of lymph nodes, tumor stage, radiotherapy and risk score, and found that miRNA signature can be used as an independent risk factor for the prognosis of gastric cancer patients (Table 1).

## Validation of the 3-miRNA signatures for survival prediction in the validation cohort

**Figure 4:**
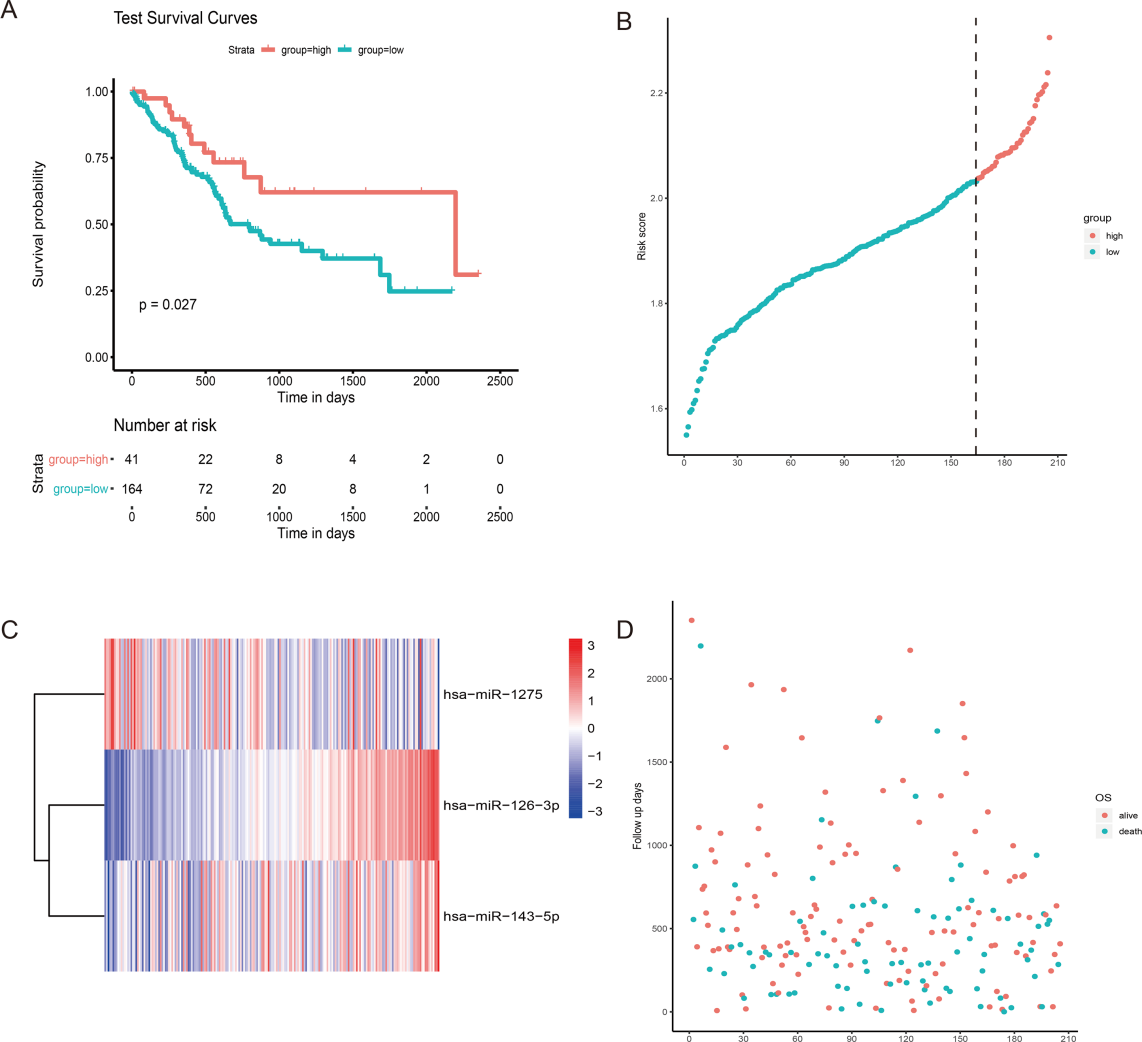
The risk score performance in the testing cohort. (A) The survival curve of 3-miRNA riskscore in testing cohort. The abscissa represents survival time (days), and the ordinate represents survival rate. The red line represents high expression and the green line represents low expression. (B) The heatmap of gene expression of three miRNA. (C) The risk score distribution of each sample. (D) Distribution of survival time for each sample.

To verify the predictive ability of the 3 miRNAs we found, we verified them in the testing cohort and found that the results were similar to the training cohort (Fig. 4), then we used univariable and multivariable cox analysis on the testing cohort to find this miRNA signature that can still be used as an independent risk factor for the GC patients prognosis (Table 2).

**Table 1:** Univariable and multivariable cox analysis of miRNA signature in training cohort.

Variable	Univariable			Multivariable		
	HR	95% CI	*p*	HR	95% CI	*p*
Age	1.426	1.03–1.974	0.032	1.416	0.98–2.046	0.064
Gender	0.92	0.674–1.256	0.600	0.895	0.628–1.276	0.540
Lymph_node	0.773	0.549–1.089	0.142	0.698	0.486–1.003	0.052
Stage	1.515	1.098–2.09	0.011	1.645	1.139–2.377	0.018
Radiation_therapy	0.441	0.254–0.767	0.014	0.518	0.295–0.908	0.022
Risk_score	0.312	0.178–0.571	<0.001	0.466	0.297–0.728	<0.001

**Table 2:** Univariable and multivariable cox analysis of miRNA signature in testing cohort.

Variable	Univariable			Multivariable		
	HR	95% CI	*p*	HR	95% CI	*p*
Age	1.210	0.889–1.646	0.225	1.137	0.809–1.599	0.46
Gender	1.164	0.827–1.638	0.383	1.251	0.864–1.812	0.236
Lymph_node	1.028	0.739–1.43	0.868	0.939	0.662–1.332	0.723
Stage	2.087	1.451–3.002	0.035	2.252	1.476–3.438	0.015
Radiation_therapy	0.562	0.357–0.883	0.013	0.477	0.291–0.782	0.231
Risk_score	0.617	0.393–0.867	<0.001	0.683	0.479–0.821	0.003

## Functional enrichment analysis of miRNA target genes

**Figure 5:**
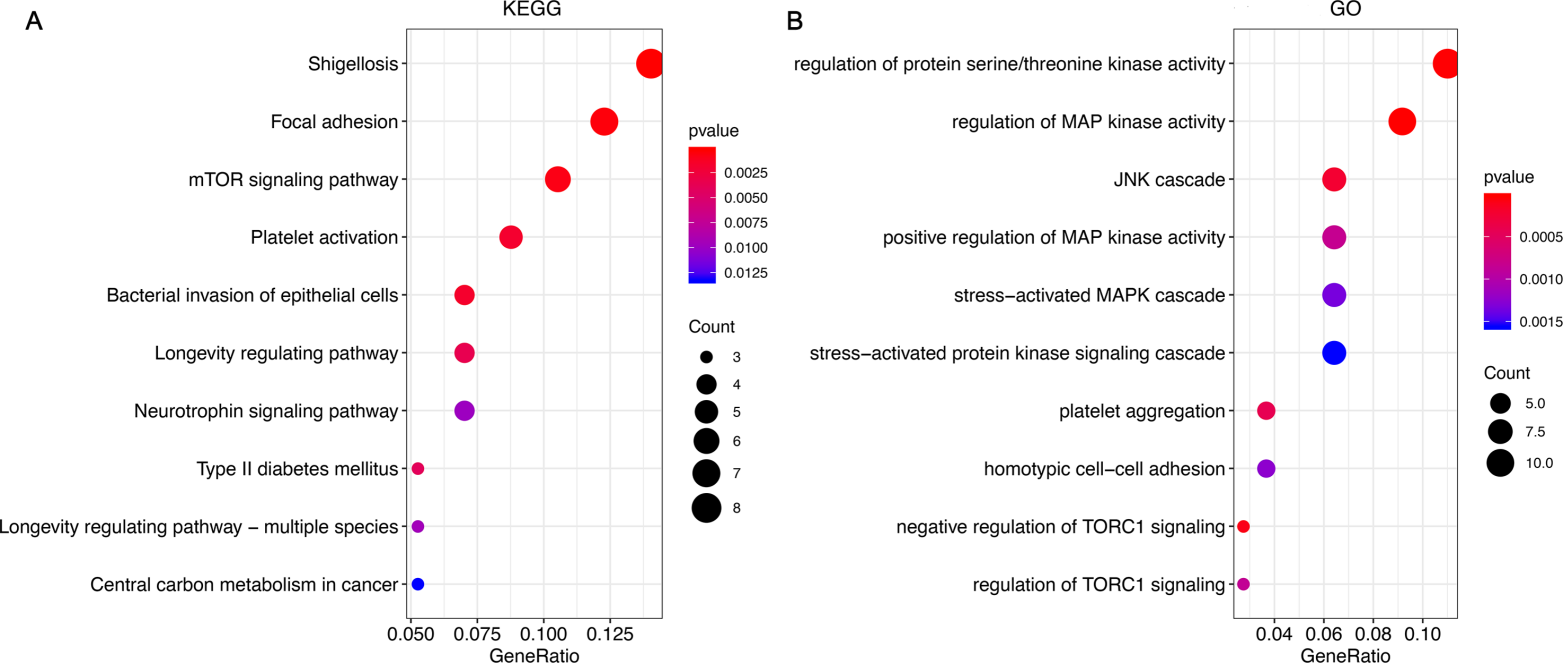
Functional enrichment analysis of miRNA target genes. (A) Pathway analysis of miRNA target genes. The abscissa represents the proportion of genes in each functional area; the ordinate represents the enriched pathway, the size of the circle represents the number of genes, the redr the color, the smaller the *P* value; (B) biological functional enrichment analysis of miRNA target genes.The abscissa represents the proportion of genes in each functional area; the ordinate represents the enriched function, the size of the circle represents the number of genes; the more red the color, the smaller the *P* value.

A total of unique 157 targeted genes regulated by three miRNAs (Table S3).The target genes are mainly enriched in tumor-related pathways such as *Focal adhesion*, *mTOR signaling pathway*, *Bacterial invasion of epithelial cells* and so on. In terms of GO function, it is mainly enriched in regulation of *MAP kinase activity*, *negative regulation of TORC1 signaling*, *homotypic cell–cell adhesion*, *stress-activated MAPK cascade* and so on (Fig. 5).

## Construct nomogram and model evaluation

**Figure 6:**
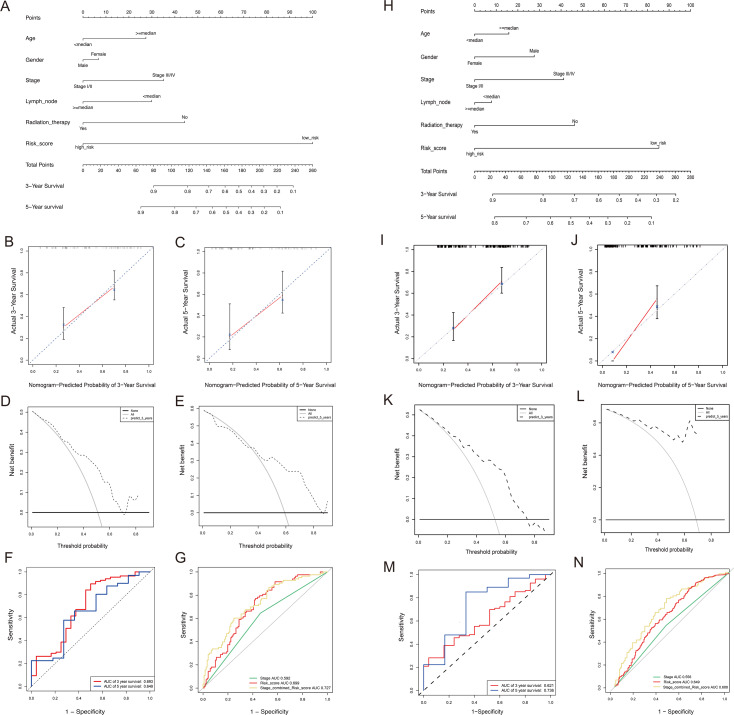
Construct nomogram and model evaluation. (A) The nomogram to predict 3-year and 5-year OS in training cohort. (B–C) The calibration plots for predicting patient 3-year and 5-year OS. (D–E) DCA for assessment of the clinical utility of the nomogram in 3-year and 5-year. The *x*-axis represents the percentage of threshold probability, and the *y*-axis represents the net benefit. (F) ROC curve based on the nomogram for 3-year and 5-year OS probability. (G) ROC analysis of the sensitivity and specificity of the survival prediction by the 3-miRNA risk score, TNM stage in training cohort. (H) The nomogram to predict 3-year and 5-year OS in testing cohort. (I–J) The calibration plots for predicting patient 3-year and 5-year OS. (K–L) DCA for assessment of the clinical utility of the nomogram in 3-year and 5-year. The *x*-axis represents the percentage of threshold probability, and the *y*-axis represents the net benefit. (M) ROC curve based on the nomogram for 3-year and 5-year OS probability. The abscissa represents false postive; the ordinate represents the true positive. (N) ROC analysis of the sensitivity and specificity of the survival prediction by the 3-miRNA risk score, TNM stage in testing cohort. The abscissa represents false postive; the ordinate represents the true positive.

We found that Riskscore has the longest line in training cohort, indicating it is the most significant for nomograms (Fig. 6A). Calibration plots were used to visualize the performances of the nomograms. The 45° line represented the best prediction. Calibration plots showed that the nomogram performed well (Figs. 6B–6C). The clinical usefulness was assessed using DCA. The nomogram showed the best net benefit (Figs. 6D–6E).

We used the ROC curve to evaluate the accuracy of the nomogram at different times. We found that the AUC for 3 years was 0.69, and for 5 years is 0.65 (Fig. 6F). We know that TNM staging plays an important role in the occurrence and development of diseases. Therefore, we further explored the impact of stage and the miRNA signature on GC survival. ROC analysis was evaluated, and as shown in Fig. 6G, we found that the ROC of the TNM stage was 0.59, and the ROC of the 3-miRNA risk score was 0.69.

Combined with the prediction of 3-miRNA signature and TNM, the AUC area reached 0.72. When 07 ≤AUC ≤0.8, it is within the range of acceptable discrimination. This shows that our prognostic model, combined with TNM, will have better survival prediction abilities and clinical utility. The results of the testing cohort are consistent with the training cohort, proving the stability of our signature (Figs. 6H–6M).

## Comparison of other signatures in GC

By consulting the literature, we selected two prognostic risk models: three-miRNA signature (Zhang et al., 2018), five-miRNA signature (Zhang et al., 2019), and our miRNAs model for comparison. To make the models comparable, we calculated the risk score of each sample in the training cohort using the same method according to the corresponding miRNAs in these two models.

**Figure 7:**
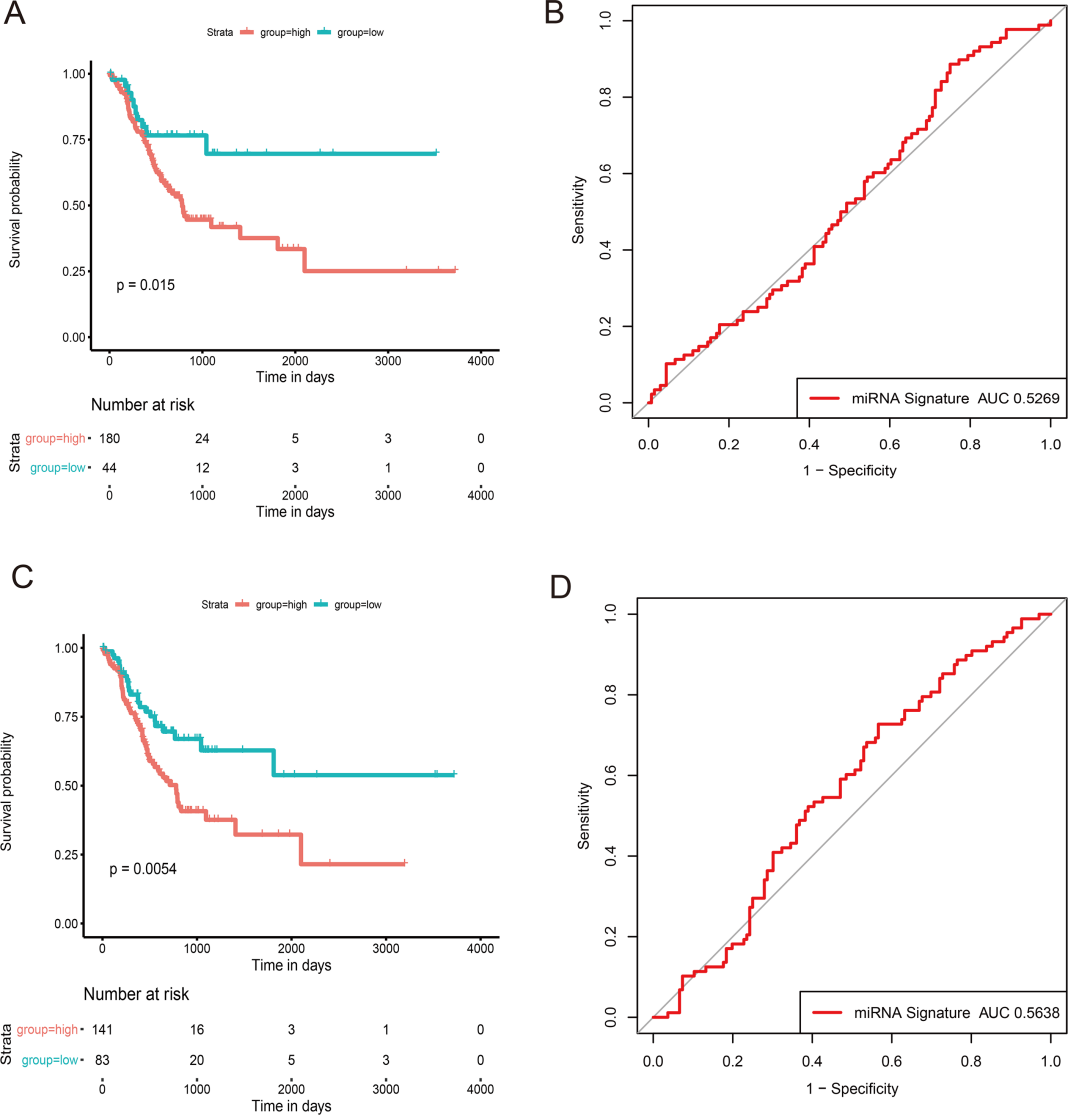
Comparison of other signatures in GC. (A) The survival curve and ROC curve in 3-miRNA signature; (B) The survival curve and ROC curve in 5-miRNA signature. The abscissa represents survival time (days), and the ordinate represents survival rate. The red line represents high expression and the green line represents low expression. The abscissa represents false postive; the ordinate represents the true positive.

Then, we evaluated the ROC of each model and calculated the prognostic difference between the high- and low-risk groups. Although the two miRNA models can significantly distinguish the prognosis, all the AUC curve <0.6, which is lower than our miRNA model (Fig. 7).

## Validation of miRNA expression in clinical samples

**Figure 8:**
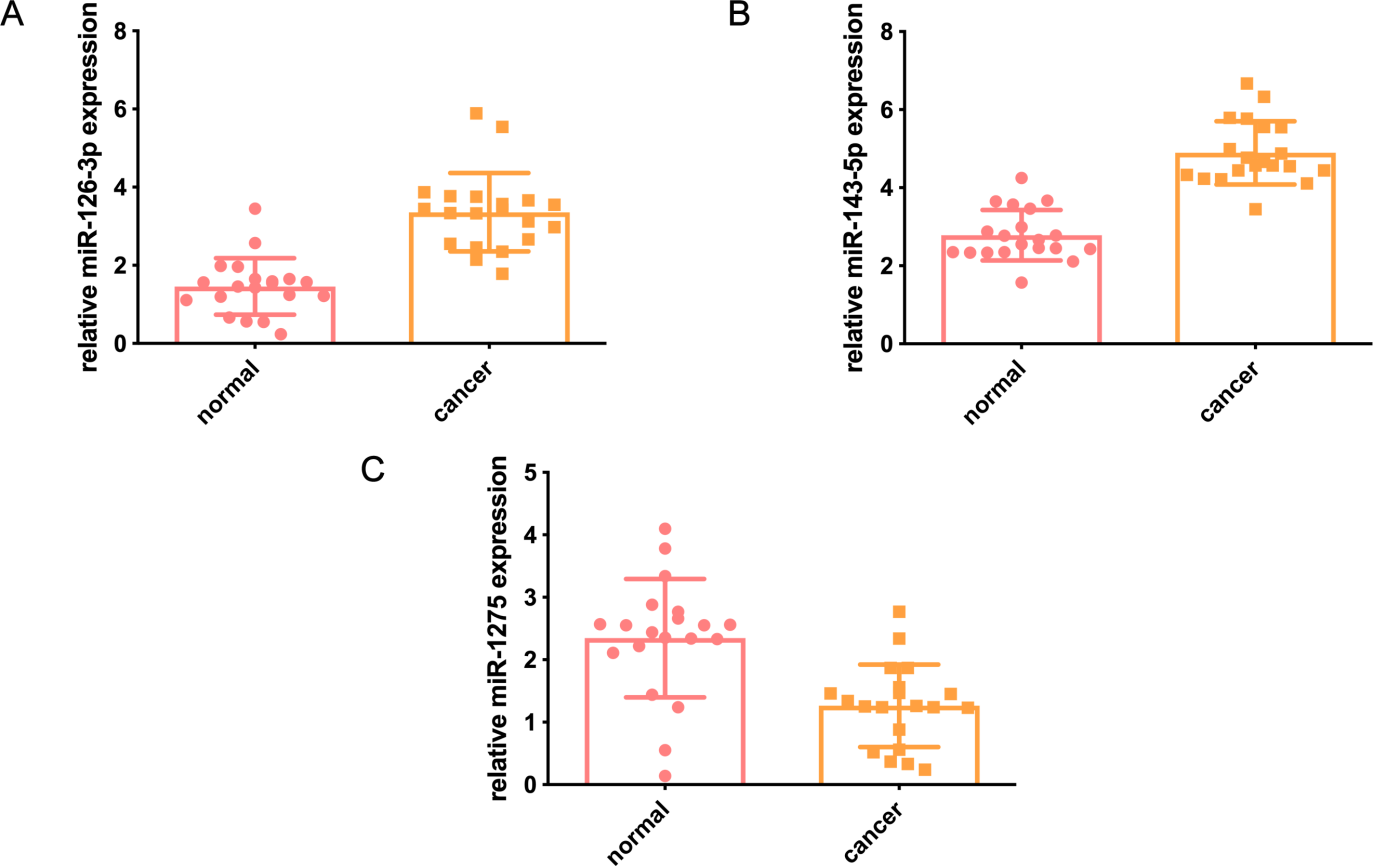
Verification of miRNA expression in clinical samples. (A) The expression of hsa-miR-126-3p in 20 pairs of GC and tumor-adjacent normal tissues. (B) The expression of hsa-miR-143-5p in GC and tumor-adjacent normal tissues. (C) The expression of hsa-miR-127 in GC and tumor-adjacent normal tissues. The abscissa represents the tissue type, and the ordinate represents the expression level of miRNA.

Twenty pairs of GC and tumor-adjacent normal tissues were included. We found that hsa-miR-126-3p and hsa-miR-143-5p are highly expressed in gastric cancer tissues compared to adjacent normal tissues, and hsa-miR-1275 is lowly expressed in tumor tissues compared to adjacent normal tissues. The expression trend is consistent with our training cohort and validation cohort (Fig. 8).

## Discussion

GC is the fourth most common and deadly cancer in the world. It is also the second most common cause of cancer-related death in China (Chen et al., 2016; Torre et al., 2015). Surgery occupies a rather important role for patients with advanced gastric cancer. In some countries, the treatment mode of gastric cancer has been developed from a single surgical treatment to perioperative chemotherapy, coupled with standardized surgery (Qi et al., 2016). Distinguishing the high-risk GC patients with poor prognosis and determine the most appropriate individualized treatment is critical.

miRNA is a type of small RNA of non-coding protein with a length of 20 to 25 nucleotides. They have demonstrated involvement in a variety of physiological and pathological processes, including cell proliferation and differentiation, invasion, and apoptosis, as well as cell cycle regulation. Abnormal expression of miRNA is essential for the progression of GC.

A total of three hub miRNAs were identified in this study. Current research shows that miR-143 generally acts as a tumor suppressor in GC and can play a role by inhibiting the migration, metastasis, and invasion of GC cells (Lei et al., 2017; Zhang et al., 2017; Wu et al., 2013). Belian et al. (2010) studied the effect of YB-1 on the conversion of miRNA expression in drug-sensitive GC and found that miR-1275 expression was up-regulated after inhibiting YB-1 expression. Mei et al. (2019) found that MicroRNA-1275 can regulate vimentin and E-cadherin through JAZF1, thereby inhibiting cell migration and invasion in GC. Feng et al. (2018) showed that down-regulated serum miR-126 is associated with aggressive progression and poor prognosis of gastric cancer. Li, Wang & Wang (2018) found that miR-126 functions as a Tumor Suppressor by Targeting SRPK1 in Human Gastric Cancer. However, it has not been reported that the combination of these 3 miRNAs can predict the prognosis of patients with GC.

In this study, we first identified the differentially expressed miRNA through GEO cohorts, then performed univariable Cox analysis on these miRNAs in the TCGA cohort. We constructed a 3-miRNA signature in the TCGA training cohort by LASSO regression analysis, and an internal testing cohort was used to prove our model has good robustness. At the same time, to prove the superiority of our signature, after consulting the literature, we finally chose the signatures of Zhang C (Wickham, 2016) and Zhang Z (Raymond et al., 2005), to compare with our miRNA model. It can be seen that although the two miRNA models can significantly distinguish the prognosis, all the AUC curves <0.6, which is lower than our miRNA model. This further shows the advantages of our model in prognosis prediction.

Pathological staging is a key prognostic factor for oncologists and GC patients. However, patients at the same cancer stage may have different clinical outcomes, indicating that the current clinical staging system is not accurate enough to distinguish the prognosis. The current pathological diagnosis is based on the anatomical structure and staging system of the disease, and cannot fully reflect the biological heterogeneity of GC patients. These problems may affect the accuracy of traditional systems in predicting GC patient prognosis.

We combined the 3-miRNA signatures to construct the nomogram. DCA and calibration were used to confirm that the risk model could perform a reliable and satisfactory prediction of patient prognosis. We found that compared with the traditional pathological stage, the nomogram has the best ability to predict the prognosis.

## Conclusion

We established a novel 3-miRNA prognostic risk model for GC. The results show that the 3-miRNA prognosis model is a reliable tool for predicting the prognosis of GC. The nomogram can help clinicians choose personalized treatment for GC patients. However, large-scale prospective and multi-center studies are still needed to evaluate the robustness of the model before clinical application. The potential biological mechanisms associated with the model should also be studied.

##  Supplemental Information

10.7717/peerj.10462/supp-1 Table S1 A total of 171 differential expression miRNAs in the GSE23739 dataset

10.7717/peerj.10462/supp-2 Table S2 116 differential expression miRNAs in the GSE93415 dataset

10.7717/peerj.10462/supp-3 Table S3 The target gene regulated by miRNA

10.7717/peerj.10462/supp-4 Figure S1 The target gene expression regulated by miRNA

## Abbreviations

GEO: Gene Expression Omnibus
KEGG: Kyoto Encyclopedia of Genes and Genomes
GO: Gene Oncology
GC: Gastric cancer
ROC: The receiver operating characteristic curve
TCGA: The Cancer Genome Atlas
DCA: Decision curve analysis

## Additional Information and Declarations

### Data Availability

The following information was supplied regarding data availability: Data are available in the Supplemental Files. Additional data is available at NCBI GEO: GSE23739 and GSE93415.
