# Supplementary material for: Development and clinical validation of a 3-miRNA signature to predict prognosis of gastric cancer
Source: PeerJ. 2021 Feb 3;9:e10462. doi: 10.7717/peerj.10462 (PMC7866890; doi:10.7717/peerj.10462)
Supplement: Table S1 [file peerj-09-10462-s001.docx]

Table S1:

| ID | logFC | AveExpr | t | P.Value | adj.P.Val | B |
| --- | --- | --- | --- | --- | --- | --- |
| hsa-miR-494 | -2.42161 | 10.18319 | -8.46471 | 6.43E-13 | 5.12E-10 | 19.0236 |
| hsa-miR-18a | 2.558949 | 5.572738 | 8.21209 | 2.08E-12 | 8.29E-10 | 17.89409 |
| hsa-miR-18b | 2.095411 | 5.571425 | 7.075644 | 3.90E-10 | 7.92E-08 | 12.86764 |
| hsa-miR-513a-5p | -2.84724 | 6.91453 | -7.0712 | 3.98E-10 | 7.92E-08 | 12.84825 |
| hsa-miR-17 | 2.409119 | 8.235546 | 7.009191 | 5.27E-10 | 8.39E-08 | 12.57817 |
| hsa-miR-923 | -1.43253 | 16.18934 | -6.79194 | 1.41E-09 | 1.86E-07 | 11.63669 |
| hsa-miR-345 | -2.74141 | 3.939098 | -6.63694 | 3.19E-09 | 3.62E-07 | 10.86011 |
| hsa-miR-513b | -3.08958 | 6.107853 | -6.34594 | 1.03E-08 | 9.66E-07 | 9.731019 |
| hsa-miR-93 | 1.54197 | 8.132931 | 6.331533 | 1.09E-08 | 9.66E-07 | 9.670156 |
| hsa-miR-21 | 2.457034 | 12.60122 | 6.233725 | 1.68E-08 | 1.34E-06 | 9.258204 |
| hsa-miR-20a | 2.783308 | 8.312001 | 6.168555 | 2.23E-08 | 1.62E-06 | 8.984983 |
| hsa-miR-513c | -2.71214 | 5.096491 | -6.09816 | 3.13E-08 | 2.08E-06 | 8.664688 |
| hsa-miR-181a | 1.265274 | 7.632246 | 6.054337 | 3.67E-08 | 2.25E-06 | 8.508676 |
| hsa-miR-301b | 1.557605 | 3.748495 | 5.920249 | 7.15E-08 | 4.07E-06 | 7.881936 |
| hsa-miR-301a | 2.184956 | 5.85859 | 5.873096 | 8.26E-08 | 4.38E-06 | 7.737185 |
| hsa-miR-15a | 2.382428 | 9.184628 | 5.806818 | 1.07E-07 | 5.31E-06 | 7.488439 |
| hsa-miR-20b | 2.256957 | 6.985268 | 5.727391 | 1.54E-07 | 7.19E-06 | 7.144189 |
| hsa-miR-7 | 2.508082 | 4.856183 | 5.712541 | 1.72E-07 | 7.33E-06 | 7.041947 |
| hsa-miR-106b | 1.362663 | 9.544718 | 5.690522 | 1.75E-07 | 7.33E-06 | 7.015165 |
| hsa-miR-17* | 1.281132 | 6.243042 | 5.640972 | 2.16E-07 | 8.40E-06 | 6.814775 |
| hsa-miR-16 | 2.72614 | 9.693914 | 5.634871 | 2.22E-07 | 8.40E-06 | 6.790154 |
| hsa-miR-135b | 2.405194 | 2.892156 | 5.692228 | 2.44E-07 | 8.44E-06 | 6.74825 |
| hsa-miR-27a | 2.132581 | 10.6395 | 5.620102 | 2.36E-07 | 8.44E-06 | 6.730601 |
| hsa-miR-214 | 2.422575 | 7.294188 | 5.492311 | 4.03E-07 | 1.34E-05 | 6.218267 |
| hsa-miR-125a-3p | -1.5778 | 6.053715 | -5.46337 | 4.55E-07 | 1.45E-05 | 6.102979 |
| hsa-let-7i | 2.422712 | 8.99746 | 5.451443 | 4.78E-07 | 1.46E-05 | 6.055565 |
| hsa-miR-19a | 2.021994 | 8.013714 | 5.438521 | 5.05E-07 | 1.49E-05 | 6.004237 |
| hsa-miR-19b-1* | 1.549025 | 2.850827 | 5.379357 | 7.74E-07 | 2.20E-05 | 5.633613 |
| hsa-miR-146b-5p | 2.36583 | 6.064281 | 5.330735 | 8.59E-07 | 2.36E-05 | 5.516051 |
| hsa-miR-103 | 1.874 | 8.934079 | 5.262326 | 1.05E-06 | 2.78E-05 | 5.310231 |
| hsa-miR-331-3p | 1.756297 | 7.188486 | 5.096 | 2.06E-06 | 5.29E-05 | 4.665575 |
| hsa-let-7d | 2.435551 | 7.713416 | 5.079896 | 2.20E-06 | 5.40E-05 | 4.603721 |
| hsa-miR-192 | 2.524449 | 8.656673 | 5.066282 | 2.32E-06 | 5.40E-05 | 4.551513 |
| hsa-miR-425 | 1.436172 | 6.165403 | 5.065923 | 2.33E-06 | 5.40E-05 | 4.550139 |
| hsa-miR-19b | 1.291365 | 10.34419 | 5.060997 | 2.37E-06 | 5.40E-05 | 4.531265 |
| hsa-miR-662 | -1.40642 | 3.138963 | -4.9814 | 3.69E-06 | 8.16E-05 | 4.145624 |
| hsa-miR-107 | 2.134624 | 9.633531 | 4.931004 | 3.99E-06 | 8.59E-05 | 4.036794 |
| hsa-miR-455-3p | 2.085704 | 5.01718 | 4.902146 | 4.48E-06 | 9.38E-05 | 3.927965 |
| hsa-miR-192* | 1.553233 | 4.901767 | 4.879764 | 5.39E-06 | 0.000109 | 3.781274 |
| hsa-miR-223 | 2.279557 | 7.437327 | 4.852247 | 5.46E-06 | 0.000109 | 3.740621 |
| hsa-let-7e | 2.213058 | 7.595548 | 4.838139 | 5.77E-06 | 0.000112 | 3.687844 |
| hsa-miR-155 | 1.933237 | 5.59582 | 4.815138 | 6.51E-06 | 0.000123 | 3.583581 |
| hsa-miR-141* | 1.639345 | 3.535595 | 4.815617 | 6.92E-06 | 0.000128 | 3.545544 |
| hsa-miR-23a | 1.957444 | 10.44172 | 4.783745 | 7.14E-06 | 0.000129 | 3.48517 |
| hsa-miR-24 | 1.377317 | 11.11716 | 4.664902 | 1.14E-05 | 0.000201 | 3.046912 |
| hsa-miR-324-5p | 1.68112 | 5.471267 | 4.652109 | 1.19E-05 | 0.000206 | 3.000113 |
| hsa-miR-181c | 1.421329 | 4.936239 | 4.630826 | 1.29E-05 | 0.000219 | 2.922428 |
| hsa-miR-552 | 3.011078 | 1.855749 | 4.713498 | 1.56E-05 | 0.000253 | 2.895435 |
| hsa-let-7f | 2.15667 | 9.782147 | 4.585535 | 1.54E-05 | 0.000253 | 2.757808 |
| hsa-miR-376b | 1.812253 | 3.11229 | 4.574497 | 1.72E-05 | 0.000273 | 2.681904 |
| hsa-miR-215 | 2.167833 | 7.662162 | 4.549333 | 1.79E-05 | 0.00028 | 2.620417 |
| hsa-miR-564 | -1.59561 | 3.065627 | -4.55711 | 1.86E-05 | 0.000284 | 2.612912 |
| hsa-miR-196b | 1.858694 | 3.378454 | 4.547555 | 1.98E-05 | 0.000297 | 2.562543 |
| hsa-let-7a | 1.99253 | 10.57378 | 4.515231 | 2.02E-05 | 0.000297 | 2.504186 |
| hsa-miR-34a | 1.85839 | 9.294188 | 4.46842 | 2.41E-05 | 0.000349 | 2.336627 |
| hsa-miR-590-5p | 1.701761 | 5.277088 | 4.458173 | 2.59E-05 | 0.000369 | 2.282813 |
| hsa-miR-96 | 2.261687 | 5.896274 | 4.417987 | 2.95E-05 | 0.000411 | 2.151983 |
| hsa-miR-29b-1* | 1.438178 | 3.052644 | 4.355053 | 3.99E-05 | 0.000548 | 1.90039 |
| hsa-miR-370 | -1.36727 | 4.256632 | -4.30165 | 4.54E-05 | 0.000613 | 1.744156 |
| ebv-miR-BART19-3p | -2.38472 | 4.052065 | -4.30099 | 4.81E-05 | 0.000638 | 1.719338 |
| hsa-miR-200a | 1.950132 | 9.489429 | 4.267386 | 5.10E-05 | 0.000663 | 1.629375 |
| hsa-miR-25 | 1.192645 | 6.994865 | 4.262491 | 5.20E-05 | 0.000663 | 1.61241 |
| hsa-miR-185 | 1.117167 | 6.755808 | 4.259843 | 5.25E-05 | 0.000663 | 1.603239 |
| hsa-miR-708 | -1.43244 | 2.825539 | -4.25799 | 5.50E-05 | 0.000684 | 1.579822 |
| hsa-miR-29b | 1.759584 | 10.97963 | 4.240305 | 5.64E-05 | 0.000691 | 1.535677 |
| hsa-miR-181a-2* | 1.485718 | 1.447915 | 4.255272 | 7.68E-05 | 0.000913 | 1.405069 |
| hsa-miR-200b | 1.884647 | 8.189038 | 4.182455 | 6.97E-05 | 0.000841 | 1.336812 |
| hsa-miR-509-5p | -1.81307 | 1.256078 | -4.20077 | 8.25E-05 | 0.000938 | 1.298666 |
| hsa-miR-454 | 1.48761 | 2.846963 | 4.156636 | 8.19E-05 | 0.000938 | 1.223108 |
| hsa-miR-629* | 1.807181 | 2.391235 | 4.165823 | 8.81E-05 | 0.000974 | 1.214273 |
| hsa-miR-199a-3p | 2.527501 | 7.493216 | 4.134284 | 8.45E-05 | 0.000948 | 1.166522 |
| hsa-miR-221 | 1.525232 | 5.941083 | 4.09528 | 9.56E-05 | 0.001042 | 1.040516 |
| hsa-miR-142-3p | 2.249172 | 10.22451 | 4.057355 | 0.00011 | 0.001179 | 0.912902 |
| hsa-miR-502-5p | 1.237243 | 3.273882 | 4.052723 | 0.000117 | 0.001221 | 0.880972 |
| hsa-miR-98 | 1.930187 | 4.359691 | 4.042404 | 0.00012 | 0.001221 | 0.853404 |
| hsa-let-7g | 1.706414 | 8.263158 | 4.033741 | 0.000119 | 0.001221 | 0.833844 |
| hsa-miR-151-5p | 1.530988 | 7.198033 | 4.014439 | 0.000128 | 0.001287 | 0.769455 |
| hsa-miR-214* | 2.024328 | 2.504059 | 4.002984 | 0.000153 | 0.001524 | 0.687677 |
| hsa-miR-27b | 1.735046 | 9.836059 | 3.933558 | 0.00017 | 0.001671 | 0.501916 |
| hsa-miR-342-5p | 1.243525 | 3.380634 | 3.924682 | 0.00018 | 0.001728 | 0.468242 |
| hsa-miR-28-5p | 1.809931 | 6.843353 | 3.917069 | 0.00018 | 0.001728 | 0.447831 |
| hsa-miR-32* | 2.115796 | 3.118911 | 3.914178 | 0.000203 | 0.001904 | 0.410632 |
| hsa-miR-199a-5p | 1.742916 | 8.722986 | 3.893025 | 0.000196 | 0.001858 | 0.369244 |
| hsa-miR-15b | 1.098938 | 7.287648 | 3.859586 | 0.00022 | 0.002016 | 0.260507 |
| hsa-miR-34b* | 1.524614 | 5.914684 | 3.846141 | 0.000236 | 0.002133 | 0.214007 |
| hsa-miR-125a-5p | 1.506932 | 4.362285 | 3.735327 | 0.00034 | 0.003043 | -0.13812 |
| hsa-miR-429 | 1.33629 | 7.343761 | 3.689069 | 0.000396 | 0.00346 | -0.2837 |
| kshv-miR-K12-7 | -1.57343 | 1.228283 | -3.69223 | 0.000475 | 0.004114 | -0.29752 |
| hsa-miR-92a-1* | 1.540095 | 1.207367 | 3.648315 | 0.000526 | 0.004454 | -0.41588 |
| hsa-miR-95 | 1.75746 | 1.967434 | 3.643183 | 0.000516 | 0.004418 | -0.42607 |
| hsa-miR-424 | 1.578617 | 6.393497 | 3.559585 | 0.000614 | 0.005035 | -0.68447 |
| hsa-miR-505* | 1.070662 | 2.368326 | 3.549114 | 0.000646 | 0.005246 | -0.71455 |
| hsa-miR-126 | 1.55599 | 6.875297 | 3.538242 | 0.000662 | 0.005301 | -0.74866 |
| hsa-miR-203 | 1.364848 | 4.34011 | 3.536397 | 0.000666 | 0.005301 | -0.7545 |
| hsa-miR-34a* | 1.125961 | 3.225312 | 3.521369 | 0.000729 | 0.005742 | -0.79449 |
| hsa-miR-873 | -1.17037 | 2.488891 | -3.49378 | 0.000817 | 0.006379 | -0.86768 |
| hsa-miR-516a-5p | -1.60705 | 3.143013 | -3.45488 | 0.000908 | 0.00695 | -0.98802 |
| hsa-miR-199b-5p | 1.792638 | 7.590102 | 3.415391 | 0.001002 | 0.007524 | -1.11175 |
| hsa-miR-10a | 1.516506 | 5.183267 | 3.416952 | 0.000977 | 0.007409 | -1.11366 |
| hsa-miR-10b* | -1.13611 | 3.025379 | -3.38051 | 0.001114 | 0.008213 | -1.21668 |
| hsa-miR-140-5p | 1.460601 | 6.707014 | 3.379168 | 0.001098 | 0.008171 | -1.22672 |
| hsa-miR-10b | 1.38857 | 3.768375 | 3.370904 | 0.001172 | 0.008408 | -1.23847 |
| hsa-miR-768-5p_v11.0 | 1.237108 | 8.397269 | 3.370836 | 0.001133 | 0.008275 | -1.24961 |
| hsa-miR-24-1* | 1.50258 | 4.603052 | 3.364707 | 0.001161 | 0.0084 | -1.26585 |
| hsa-miR-361-5p | 1.018434 | 5.906136 | 3.340091 | 0.001244 | 0.008767 | -1.34122 |
| hsa-miR-542-5p | 1.369588 | 3.540647 | 3.34002 | 0.001245 | 0.008767 | -1.34143 |
| hsa-miR-142-5p | 1.182058 | 8.079464 | 3.333238 | 0.001272 | 0.00888 | -1.3612 |
| hsa-miR-195 | 2.133992 | 7.879813 | 3.313931 | 0.001352 | 0.009358 | -1.41731 |
| hsa-miR-183 | 1.235798 | 3.767326 | 3.298187 | 0.001452 | 0.00988 | -1.45209 |
| hsa-miR-451 | 1.874299 | 10.10368 | 3.295601 | 0.001433 | 0.00983 | -1.47035 |
| hsa-miR-130a | 1.250778 | 8.804545 | 3.194169 | 0.001966 | 0.012724 | -1.75974 |
| hsa-let-7c | 1.131905 | 9.760119 | 3.181389 | 0.002045 | 0.013129 | -1.7957 |
| hsa-miR-376a* | 1.366608 | 1.851216 | 3.161294 | 0.002275 | 0.014257 | -1.82379 |
| hsa-miR-632 | -1.1812 | 1.990204 | -3.14178 | 0.002511 | 0.015143 | -1.85632 |
| hsa-miR-499-3p | 1.899672 | -0.13567 | 3.128193 | 0.003042 | 0.017394 | -1.86377 |
| hsa-miR-328 | 1.577269 | 3.207624 | 3.135635 | 0.002436 | 0.014803 | -1.90019 |
| hsa-miR-146a | 1.536061 | 7.590278 | 3.139056 | 0.002329 | 0.014483 | -1.91402 |
| hsa-miR-196a | 1.213865 | 3.689616 | 3.134241 | 0.002398 | 0.014796 | -1.91692 |
| hsa-miR-338-3p | 1.304344 | 7.922295 | 3.126019 | 0.002423 | 0.014803 | -1.9502 |
| hsa-miR-589* | -3.8451 | 0.619863 | -3.13504 | 0.004775 | 0.025859 | -1.99767 |
| hsa-let-7d* | 1.195636 | 2.601131 | 3.102908 | 0.002646 | 0.015488 | -2.00007 |
| hsa-miR-200c | 1.18173 | 9.57207 | 3.105936 | 0.002576 | 0.015417 | -2.00571 |
| hsa-miR-630 | 1.000774 | 7.504919 | 3.100652 | 0.002618 | 0.015488 | -2.02027 |
| hsa-miR-375 | -1.35783 | 8.670043 | -3.09847 | 0.002644 | 0.015488 | -2.02353 |
| hsa-miR-125b | 1.652507 | 8.345685 | 3.084784 | 0.002746 | 0.015841 | -2.06387 |
| hsa-miR-410 | 1.24416 | 2.820051 | 3.035534 | 0.003248 | 0.018338 | -2.18043 |
| hsa-miR-127-5p | -1.02611 | 1.293266 | -2.91148 | 0.004919 | 0.026454 | -2.45971 |
| hsa-miR-23b | 1.24991 | 10.0783 | 2.925124 | 0.004411 | 0.024217 | -2.49256 |
| hsa-miR-181c* | 1.060671 | 2.975335 | 2.888274 | 0.004993 | 0.026674 | -2.56545 |
| hsa-miR-224 | 1.625042 | 2.615417 | 2.828183 | 0.006187 | 0.031774 | -2.66734 |
| hsa-miR-101 | 1.053704 | 8.958705 | 2.851537 | 0.005457 | 0.028769 | -2.68391 |
| hsa-miR-150 | 1.251528 | 6.812795 | 2.839394 | 0.005679 | 0.029546 | -2.70763 |
| hsa-miR-609 | 1.273616 | 1.783926 | 2.737447 | 0.008199 | 0.040535 | -2.85887 |
| hcmv-miR-UL22A* | 1.256718 | 0.571198 | 2.691616 | 0.009868 | 0.047035 | -2.91062 |
| hsa-miR-1224-3p | 1.914133 | 1.660662 | 2.725908 | 0.008226 | 0.040535 | -2.91793 |
| hsa-miR-129-5p | -1.19224 | 1.972205 | -2.72686 | 0.008069 | 0.040396 | -2.93752 |
| hsa-miR-1 | 1.48696 | 2.814391 | 2.725748 | 0.007977 | 0.040186 | -2.96134 |
| hsa-miR-15a* | 1.040536 | 1.885379 | 2.714499 | 0.00825 | 0.040535 | -2.97437 |
| ebv-miR-BART14* | 1.809649 | 0.120461 | 2.645993 | 0.010848 | 0.050496 | -3.03364 |
| hsa-miR-126* | 1.033796 | 1.471694 | 2.683893 | 0.009097 | 0.044425 | -3.03536 |
| hsa-miR-488* | 1.64325 | 0.536717 | 2.600893 | 0.013636 | 0.060639 | -3.05094 |
| hsa-miR-650 | -1.25601 | 2.935787 | -2.66667 | 0.009328 | 0.04506 | -3.11206 |
| hsa-miR-32 | 1.126987 | 4.787428 | 2.643709 | 0.00986 | 0.047035 | -3.18135 |
| hsa-miR-125b-1* | -1.32279 | 1.479522 | -2.54267 | 0.014663 | 0.063434 | -3.20173 |
| hsa-miR-449a | 1.072193 | 1.610917 | 2.60688 | 0.011093 | 0.051338 | -3.2332 |
| hsa-miR-934 | 1.34399 | 1.533533 | 2.579611 | 0.012118 | 0.055435 | -3.26792 |
| hsa-miR-548c-3p | 1.599053 | 2.912545 | 2.506001 | 0.014601 | 0.063434 | -3.44375 |
| hsa-miR-100 | 1.262697 | 5.814515 | 2.516921 | 0.013806 | 0.061054 | -3.48417 |
| hsa-miR-139-5p | -1.13557 | 3.052758 | -2.48057 | 0.015416 | 0.066328 | -3.52772 |
| hsa-miR-377 | 1.045798 | 6.246312 | 2.497402 | 0.014434 | 0.063434 | -3.54757 |
| hcmv-miR-US25-1* | 2.079489 | 0.375511 | 2.345812 | 0.025103 | 0.097238 | -3.55936 |
| hsa-miR-99a | 1.440802 | 6.899938 | 2.468348 | 0.015621 | 0.066578 | -3.60465 |
| hsa-miR-218 | 1.230231 | 2.557048 | 2.442253 | 0.016944 | 0.070249 | -3.62488 |
| hsa-miR-26b | 1.112616 | 6.872856 | 2.442535 | 0.016728 | 0.069713 | -3.65802 |
| hsa-miR-374a | 1.025881 | 3.644103 | 2.39748 | 0.018957 | 0.076989 | -3.72275 |
| hsa-miR-181a* | 1.136806 | 1.564104 | 2.304469 | 0.024603 | 0.096473 | -3.83152 |
| hsa-miR-518c | 1.759365 | 0.280884 | 2.201784 | 0.033788 | 0.12394 | -3.87087 |
| hsa-miR-644 | -1.79637 | 0.978323 | -2.17586 | 0.035815 | 0.128078 | -3.92771 |
| hsa-miR-143 | 1.376347 | 8.77751 | 2.325034 | 0.022448 | 0.089791 | -3.93235 |
| hsa-miR-380* | 2.006486 | 0.866393 | 2.093313 | 0.045429 | 0.151304 | -3.97579 |
| hsa-miR-25* | 1.257915 | 1.263054 | 2.208445 | 0.031164 | 0.117013 | -4.00952 |
| hsa-miR-513a-3p | -1.35121 | 0.848224 | -2.14676 | 0.037336 | 0.131502 | -4.03539 |
| hsa-miR-220b | 1.453247 | 0.675302 | 2.091174 | 0.043588 | 0.147018 | -4.04614 |
| hsa-miR-493* | 1.266121 | 1.446339 | 2.188547 | 0.032655 | 0.120899 | -4.05983 |
| hsa-miR-518a-3p | 1.151974 | 0.454774 | 2.142927 | 0.036239 | 0.128205 | -4.15568 |
| hsa-miR-190b | -1.05274 | 0.690856 | -2.08582 | 0.0422 | 0.145417 | -4.19147 |
| hsa-miR-30b | 1.026243 | 6.822861 | 2.177858 | 0.032213 | 0.11982 | -4.23654 |
| hsa-miR-106b* | 1.126109 | 0.894111 | 2.111136 | 0.038785 | 0.135408 | -4.23918 |
| hsa-miR-615-3p | 1.024787 | 1.538635 | 2.089692 | 0.04012 | 0.139455 | -4.35012 |
| hiv1-miR-N367 | 1.135691 | 1.266596 | 2.026641 | 0.047288 | 0.155542 | -4.37258 |
| ebv-miR-BART11-5p | 1.116575 | 0.803118 | 2.006399 | 0.049548 | 0.160982 | -4.39714 |
