# Supplementary material for: Development and clinical validation of a 3-miRNA signature to predict prognosis of gastric cancer
Source: PeerJ. 2021 Feb 3;9:e10462. doi: 10.7717/peerj.10462 (PMC7866890; doi:10.7717/peerj.10462)
Supplement: Table S2 [file peerj-09-10462-s002.docx]

Table S2

| ID | logFC | AveExpr | t | P.Value | adj.P.Val | B |
| --- | --- | --- | --- | --- | --- | --- |
| hsa-miR-181b-5p | 1.20595 | -0.05218 | 8.903887 | 4.21E-11 | 1.77E-08 | 15.24967 |
| hsa-miR-4290 | -1.2741 | -0.29895 | -8.65969 | 8.91E-11 | 1.77E-08 | 14.52132 |
| hsa-miR-4268 | -1.2915 | -0.3807 | -8.65072 | 9.16E-11 | 1.77E-08 | 14.49444 |
| hsa-miR-4279 | -1.08415 | -0.32678 | -8.408 | 1.94E-10 | 3.09E-08 | 13.76314 |
| hsa-miR-23a-3p | 1.6759 | -0.2103 | 8.097583 | 5.14E-10 | 5.44E-08 | 12.8181 |
| hsa-miR-891a | -1.2999 | -0.275 | -7.98646 | 7.29E-10 | 6.32E-08 | 12.47726 |
| hsa-miR-181a-5p | 1.15225 | -0.03048 | 7.879247 | 1.02E-09 | 6.78E-08 | 12.14725 |
| hsa-miR-4291 | 1.49555 | -0.31588 | 7.851009 | 1.12E-09 | 6.78E-08 | 12.06014 |
| hsa-miR-1269b | -1.01665 | -0.36218 | -7.84592 | 1.14E-09 | 6.78E-08 | 12.04445 |
| hsa-miR-331-3p | 1.17655 | 0.093425 | 7.71198 | 1.74E-09 | 9.76E-08 | 11.63014 |
| hsa-miR-4539 | -1.4428 | -0.43545 | -7.61606 | 2.36E-09 | 1.19E-07 | 11.33242 |
| hsa-let-7d-3p | -1.5582 | -0.74365 | -7.13736 | 1.10E-08 | 4.36E-07 | 9.835371 |
| hsa-miR-214-3p | 1.6432 | -0.40825 | 7.103432 | 1.23E-08 | 4.67E-07 | 9.728622 |
| hsa-miR-181d | 1.02855 | 0.043825 | 7.049659 | 1.46E-08 | 4.97E-07 | 9.559311 |
| hsa-miR-660-3p | -1.34425 | -0.57338 | -6.9941 | 1.74E-08 | 5.74E-07 | 9.384199 |
| hsa-miR-92a-3p | 1.1539 | 0.0056 | 6.499778 | 8.69E-08 | 1.95E-06 | 7.819687 |
| hsa-miR-4661-5p | -1.37635 | -0.56118 | -6.48226 | 9.20E-08 | 1.95E-06 | 7.764078 |
| hsa-miR-93-5p | 1.2741 | -0.0021 | 6.420424 | 1.13E-07 | 2.11E-06 | 7.567781 |
| hsa-miR-21-3p | 1.0627 | -0.164 | 6.150133 | 2.72E-07 | 4.41E-06 | 6.709172 |
| hsa-miR-4262 | 1.29615 | -0.21213 | 6.149232 | 2.73E-07 | 4.41E-06 | 6.706311 |
| hsa-miR-196a-5p | 1.84665 | -0.12993 | 6.099206 | 3.21E-07 | 5.02E-06 | 6.547389 |
| hsa-miR-214-5p | 1.34045 | -0.06318 | 6.040862 | 3.89E-07 | 5.79E-06 | 6.362077 |
| SNORD10 | 1.8342 | -0.1626 | 6.031404 | 4.01E-07 | 5.88E-06 | 6.33204 |
| hsa-miR-3175 | 1.0534 | -0.15215 | 5.873992 | 6.70E-07 | 8.64E-06 | 5.832417 |
| hsa-miR-199a-5p | 1.9549 | -0.7238 | 5.834316 | 7.62E-07 | 9.57E-06 | 5.706594 |
| SNORD44 | 1.32985 | 0.216925 | 5.776551 | 9.20E-07 | 1.09E-05 | 5.52351 |
| hsa-miR-196b-5p | 1.3817 | 0.05315 | 5.605111 | 1.61E-06 | 1.78E-05 | 4.981025 |
| hsa-miR-4501 | -1.06575 | -0.35118 | -5.59922 | 1.64E-06 | 1.80E-05 | 4.962425 |
| hsa-miR-4317 | 1.3077 | 0.1067 | 5.572749 | 1.79E-06 | 1.90E-05 | 4.878807 |
| hsa-miR-4636 | -1.6101 | -0.44535 | -5.54886 | 1.93E-06 | 2.00E-05 | 4.803378 |
| hsa-miR-665 | -1.04775 | -0.36248 | -5.428 | 2.86E-06 | 2.79E-05 | 4.422467 |
| hsa-miR-1298 | -1.3332 | -0.71075 | -5.41439 | 2.99E-06 | 2.88E-05 | 4.379634 |
| hsa-let-7i-3p | 1.1308 | 0.05475 | 5.317161 | 4.10E-06 | 3.59E-05 | 4.074166 |
| hsa-miR-4797-5p | 1.12 | -0.4229 | 5.269808 | 4.78E-06 | 4.07E-05 | 3.925708 |
| hsa-let-7i-5p | 1.35135 | -0.08113 | 5.199231 | 6.00E-06 | 4.93E-05 | 3.704862 |
| hsa-miR-24-3p | 1.4939 | -0.11455 | 5.192731 | 6.13E-06 | 4.95E-05 | 3.68455 |
| hsa-miR-335-5p | 1.161 | 0.45765 | 5.155909 | 6.90E-06 | 5.27E-05 | 3.56957 |
| hsa-miR-127-3p | 1.0754 | -0.34665 | 5.150794 | 7.02E-06 | 5.27E-05 | 3.553609 |
| hsa-miR-455-3p | 1.13355 | 0.287125 | 5.146634 | 7.11E-06 | 5.27E-05 | 3.540633 |
| hsa-miR-223-3p | 1.88165 | -0.39998 | 5.131247 | 7.47E-06 | 5.44E-05 | 3.492649 |
| hsa-miR-18a-5p | 1.1581 | 0.1148 | 5.112016 | 7.95E-06 | 5.70E-05 | 3.432714 |
| hsa-miR-23c | 1.37485 | -0.26633 | 5.056278 | 9.51E-06 | 6.72E-05 | 3.259268 |
| hsa-miR-135b-5p | 1.6218 | -0.27285 | 5.030099 | 1.03E-05 | 7.09E-05 | 3.177943 |
| hsa-miR-103a-3p | 1.0812 | 0.0872 | 5.012969 | 1.09E-05 | 7.40E-05 | 3.124778 |
| hsa-miR-18b-5p | 1.1835 | 0.2079 | 4.974649 | 1.24E-05 | 8.13E-05 | 3.005988 |
| hsa-miR-21-5p | 1.74065 | -0.00658 | 4.946163 | 1.35E-05 | 8.61E-05 | 2.917817 |
| hsa-miR-106a-5p | 1.3585 | 0.115 | 4.944363 | 1.36E-05 | 8.61E-05 | 2.912252 |
| hsa-miR-301a-3p | 1.13955 | 0.073825 | 4.944239 | 1.36E-05 | 8.61E-05 | 2.911867 |
| hsa-miR-4791 | 1.1992 | 0.29045 | 4.936529 | 1.40E-05 | 8.77E-05 | 2.888024 |
| hsa-miR-106b-5p | 1.03365 | 0.144575 | 4.928825 | 1.43E-05 | 8.93E-05 | 2.864212 |
| hsa-miR-148a-3p | -1.1978 | -0.2746 | -4.90888 | 1.53E-05 | 9.39E-05 | 2.802592 |
| hsa-miR-99b-5p | 1.0711 | -0.2666 | 4.89722 | 1.58E-05 | 9.46E-05 | 2.766607 |
| hsa-miR-10b-5p | 1.5553 | -0.1174 | 4.85274 | 1.83E-05 | 0.000105 | 2.629499 |
| hsa-miR-27b-3p | 1.5312 | -0.17745 | 4.832693 | 1.95E-05 | 0.000111 | 2.567806 |
| hsa-miR-377-3p | 1.0685 | -0.06985 | 4.797292 | 2.18E-05 | 0.000123 | 2.459025 |
| hsa-miR-10a-5p | 1.6371 | -0.26075 | 4.776782 | 2.33E-05 | 0.00013 | 2.396096 |
| hsa-miR-454-3p | 1.0083 | 0.9854 | 4.771557 | 2.36E-05 | 0.000132 | 2.380077 |
| hsa-let-7a-3p | 1.0297 | 0.32905 | 4.766509 | 2.40E-05 | 0.000133 | 2.364603 |
| hsa-miR-943 | -1.09755 | -0.61068 | -4.75986 | 2.45E-05 | 0.000135 | 2.344225 |
| hsa-miR-125b-5p | 1.98925 | -1.10953 | 4.698456 | 2.98E-05 | 0.000158 | 2.156449 |
| hsa-miR-4795-3p | -1.32 | -0.3668 | -4.68937 | 3.07E-05 | 0.00016 | 2.128708 |
| hsa-miR-17-5p | 1.48005 | 0.094125 | 4.618811 | 3.84E-05 | 0.000188 | 1.913918 |
| hsa-miR-4324 | 1.42875 | -0.70058 | 4.612995 | 3.91E-05 | 0.00019 | 1.896254 |
| hsa-miR-23b-3p | 1.7481 | -0.07185 | 4.554392 | 4.70E-05 | 0.000219 | 1.718654 |
| hsa-miR-143-5p | 1.47935 | -0.91833 | 4.473323 | 6.06E-05 | 0.000271 | 1.474143 |
| hsa-miR-151a-5p | 1.0909 | 0.16805 | 4.446207 | 6.60E-05 | 0.000289 | 1.392677 |
| hsa-miR-708-5p | 1.1161 | 0.1803 | 4.437202 | 6.78E-05 | 0.000293 | 1.365659 |
| hsa-miR-199a-3p | 2.15675 | -0.45538 | 4.423454 | 7.08E-05 | 0.000301 | 1.324445 |
| hsa-miR-199b-3p | 2.15675 | -0.45538 | 4.423454 | 7.08E-05 | 0.000301 | 1.324445 |
| hsa-miR-28-5p | 1.1807 | 0.1834 | 4.420942 | 7.14E-05 | 0.000301 | 1.316921 |
| hsa-miR-151a-5p | 1.0133 | 0.13 | 4.411486 | 7.35E-05 | 0.000309 | 1.288603 |
| hsa-miR-151b | 1.0133 | 0.13 | 4.411486 | 7.35E-05 | 0.000309 | 1.288603 |
| hsa-miR-193a-3p | 1.2192 | -0.1695 | 4.404388 | 7.52E-05 | 0.000315 | 1.267363 |
| hsa-miR-20a-5p | 1.6848 | 0.3066 | 4.307115 | 0.000102 | 0.000399 | 0.977463 |
| hsa-miR-4770 | 1.04695 | 0.004425 | 4.304503 | 0.000103 | 0.000401 | 0.969711 |
| hsa-miR-95 | 1.42215 | -0.18058 | 4.29379 | 0.000106 | 0.000408 | 0.937931 |
| hsa-miR-642b-3p | 1.1205 | -0.58705 | 4.285813 | 0.000109 | 0.000416 | 0.914287 |
| hsa-miR-27a-3p | 1.82315 | -0.14143 | 4.277524 | 0.000111 | 0.000425 | 0.889733 |
| hsa-miR-652-5p | -1.0006 | -0.0936 | -4.21635 | 0.000135 | 0.000481 | 0.709069 |
| hsa-miR-100-5p | 1.7058 | -0.8586 | 4.093193 | 0.000196 | 0.000671 | 0.348433 |
| SNORD49A | 1.4573 | -0.1545 | 4.02606 | 0.000241 | 0.000801 | 0.153662 |
| hsa-miR-199b-5p | 1.58665 | -0.01423 | 3.963936 | 0.000291 | 0.000933 | -0.02537 |
| hsa-miR-1275 | -1.20965 | -0.48523 | -3.90396 | 0.000348 | 0.001061 | -0.19707 |
| hsa-miR-20b-5p | 1.5978 | 0.37135 | 3.889129 | 0.000364 | 0.001102 | -0.23934 |
| hsa-miR-145-3p | 1.4278 | -0.57135 | 3.867324 | 0.000389 | 0.001167 | -0.30137 |
| hsa-miR-130a-3p | 1.51825 | -0.16053 | 3.824631 | 0.000441 | 0.001284 | -0.42236 |
| hsa-let-7d-5p | 1.1829 | 0.18295 | 3.811815 | 0.000459 | 0.001322 | -0.45856 |
| hsa-miR-218-5p | 1.1708 | 0.15445 | 3.793538 | 0.000484 | 0.001387 | -0.51009 |
| hsa-miR-342-3p | 1.0393 | -0.10135 | 3.789417 | 0.00049 | 0.0014 | -0.52169 |
| kshv-miR-K12-12-3p | -1.00195 | -0.64323 | -3.78659 | 0.000494 | 0.001405 | -0.52964 |
| hsa-miR-374c-5p | 1.20905 | 0.063275 | 3.774426 | 0.000512 | 0.001451 | -0.56384 |
| hsa-miR-98-5p | 1.3647 | 0.2046 | 3.753746 | 0.000545 | 0.001516 | -0.62186 |
| hsa-miR-145-5p | 1.483 | -1.07485 | 3.689213 | 0.000659 | 0.001761 | -0.80194 |
| hsa-miR-19a-3p | 1.0951 | 0.0595 | 3.680141 | 0.000677 | 0.001793 | -0.82713 |
| hsa-miR-374b-5p | 1.00765 | 0.155175 | 3.625448 | 0.000794 | 0.00207 | -0.97836 |
| hsa-miR-99a-5p | 1.60425 | -0.89298 | 3.510887 | 0.001107 | 0.00278 | -1.2914 |
| hsa-miR-4328 | 1.45485 | -1.04453 | 3.460443 | 0.00128 | 0.003091 | -1.42756 |
| hsa-let-7f-5p | 1.47695 | 0.715175 | 3.438623 | 0.001362 | 0.003265 | -1.48613 |
| hsa-miR-15a-5p | 1.10745 | 0.210625 | 3.255476 | 0.002284 | 0.005016 | -1.96961 |
| hsa-miR-142-3p | 1.5088 | -0.0553 | 3.20081 | 0.002658 | 0.005698 | -2.11097 |
| hsa-miR-1 | 1.6699 | 0.20575 | 3.196434 | 0.00269 | 0.005741 | -2.12223 |
| hsa-miR-140-5p | 1.25455 | -0.05153 | 3.185137 | 0.002775 | 0.005871 | -2.15124 |
| hsa-miR-126-5p | 1.35535 | 0.481675 | 3.177507 | 0.002834 | 0.005982 | -2.1708 |
| hsa-miR-375 | -1.06525 | -0.21598 | -3.12203 | 0.0033 | 0.006861 | -2.31219 |
| hsa-miR-133a | 1.0509 | -0.5273 | 3.106804 | 0.00344 | 0.00712 | -2.35073 |
| hsa-miR-126-3p | 1.51835 | 0.081425 | 3.044479 | 0.004074 | 0.00827 | -2.50728 |
| hsa-miR-374a-5p | 1.3449 | 0.4809 | 2.996838 | 0.004631 | 0.00911 | -2.62562 |
| hsa-let-7g-5p | 1.16625 | 0.099725 | 2.929455 | 0.005542 | 0.01066 | -2.79096 |
| hsa-miR-32-5p | 1.01255 | 0.722825 | 2.906538 | 0.005888 | 0.011201 | -2.84664 |
| hsa-let-7a-5p | 1.48995 | 0.158275 | 2.906172 | 0.005894 | 0.011201 | -2.84753 |
| hsa-miR-143-3p | 1.4245 | -1.2289 | 2.875185 | 0.006395 | 0.012009 | -2.92236 |
| hsa-miR-4284 | 1.34255 | -0.27488 | 2.684349 | 0.010462 | 0.018414 | -3.37131 |
| hsa-miR-1297 | 1.10515 | 0.547275 | 2.654904 | 0.011269 | 0.019618 | -3.43869 |
| hsa-miR-195-5p | 1.2995 | -0.1948 | 2.535062 | 0.015183 | 0.025368 | -3.70751 |
| hsa-miR-26b-5p | 1.16125 | 0.067075 | 2.345202 | 0.023974 | 0.038569 | -4.11474 |
| hsa-miR-16-5p | 1.19915 | 0.144525 | 2.344005 | 0.024042 | 0.038609 | -4.11723 |
