# Supplementary material for: Development and clinical validation of a 3-miRNA signature to predict prognosis of gastric cancer
Source: PeerJ. 2021 Feb 3;9:e10462. doi: 10.7717/peerj.10462 (PMC7866890; doi:10.7717/peerj.10462)
Supplement: Table S3 [file peerj-09-10462-s003.docx]

| miRNA | Target.Gene |
| --- | --- |
| hsa-miR-143-5p | AASDHPPT |
| hsa-miR-143-5p | AASDHPPT |
| hsa-miR-143-5p | GLCE |
| hsa-miR-143-5p | RAB12 |
| hsa-miR-143-5p | ZNF460 |
| hsa-miR-143-5p | ZNF460 |
| hsa-miR-143-5p | ZNF460 |
| hsa-miR-143-5p | TAX1BP1 |
| hsa-miR-143-5p | TAX1BP1 |
| hsa-miR-143-5p | MAT2A |
| hsa-miR-143-5p | MAT2A |
| hsa-miR-143-5p | MAT2A |
| hsa-miR-143-5p | MTFR1 |
| hsa-miR-143-5p | HAS3 |
| hsa-miR-143-5p | ZBTB18 |
| hsa-miR-143-5p | TFDP1 |
| hsa-miR-143-5p | GLRX5 |
| hsa-miR-143-5p | THRA |
| hsa-miR-143-5p | MYLIP |
| hsa-miR-143-5p | ZNF138 |
| hsa-miR-143-5p | ZNF138 |
| hsa-miR-143-5p | SLC36A1 |
| hsa-miR-143-5p | RAD21 |
| hsa-miR-143-5p | RAD21 |
| hsa-miR-143-5p | RAD21 |
| hsa-miR-143-5p | PAQR5 |
| hsa-miR-143-5p | PAQR5 |
| hsa-miR-143-5p | PAQR5 |
| hsa-miR-143-5p | PAQR5 |
| hsa-miR-143-5p | PAQR5 |
| hsa-miR-143-5p | PAQR5 |
| hsa-miR-143-5p | PAQR5 |
| hsa-miR-143-5p | PAQR5 |
| hsa-miR-143-5p | PAQR5 |
| hsa-miR-143-5p | PAQR5 |
| hsa-miR-143-5p | PAQR5 |
| hsa-miR-143-5p | PAQR5 |
| hsa-miR-143-5p | NETO2 |
| hsa-miR-143-5p | NETO2 |
| hsa-miR-143-5p | MEMO1 |
| hsa-miR-143-5p | ANKRD11 |
| hsa-miR-143-5p | MGRN1 |
| hsa-miR-143-5p | LAPTM4A |
| hsa-miR-143-5p | LAPTM4A |
| hsa-miR-143-5p | LAPTM4A |
| hsa-miR-143-5p | LAPTM4A |
| hsa-miR-143-5p | LAPTM4A |
| hsa-miR-143-5p | LAPTM4A |
| hsa-miR-143-5p | OSBPL10 |
| hsa-miR-143-5p | OSBPL10 |
| hsa-miR-143-5p | OSBPL10 |
| hsa-miR-143-5p | OSBPL10 |
| hsa-miR-143-5p | OSBPL10 |
| hsa-miR-143-5p | OSBPL10 |
| hsa-miR-143-5p | OSBPL10 |
| hsa-miR-143-5p | OSBPL10 |
| hsa-miR-143-5p | ZNF85 |
| hsa-miR-143-5p | ZNF85 |
| hsa-miR-143-5p | ZNF85 |
| hsa-miR-143-5p | ZNF117 |
| hsa-miR-143-5p | TBPL1 |
| hsa-miR-143-5p | TBPL1 |
| hsa-miR-143-5p | POFUT1 |
| hsa-miR-143-5p | POFUT1 |
| hsa-miR-143-5p | G3BP2 |
| hsa-miR-143-5p | G3BP2 |
| hsa-miR-143-5p | G3BP2 |
| hsa-miR-143-5p | DYRK1A |
| hsa-miR-143-5p | DYRK1A |
| hsa-miR-143-5p | DYRK1A |
| hsa-miR-143-5p | BMT2 |
| hsa-miR-143-5p | BMT2 |
| hsa-miR-143-5p | BMT2 |
| hsa-miR-143-5p | TOMM70 |
| hsa-miR-143-5p | TOMM70 |
| hsa-miR-143-5p | TOMM70 |
| hsa-miR-143-5p | KPNA4 |
| hsa-miR-143-5p | KPNA4 |
| hsa-miR-143-5p | ZNF772 |
| hsa-miR-143-5p | ZNF772 |
| hsa-miR-143-5p | ZNF772 |
| hsa-miR-143-5p | ABCF1 |
| hsa-miR-143-5p | ABCF1 |
| hsa-miR-143-5p | SLC25A46 |
| hsa-miR-143-5p | LUZP1 |
| hsa-miR-143-5p | ST3GAL5 |
| hsa-miR-143-5p | SESN3 |
| hsa-miR-143-5p | KBTBD2 |
| hsa-miR-143-5p | ZNF264 |
| hsa-miR-143-5p | ABL2 |
| hsa-miR-143-5p | PHLDA3 |
| hsa-miR-143-5p | PHLDA3 |
| hsa-miR-143-5p | ZFP14 |
| hsa-miR-143-5p | ZFP14 |
| hsa-miR-143-5p | KLK2 |
| hsa-miR-143-5p | ORAI2 |
| hsa-miR-143-5p | ORAI2 |
| hsa-miR-143-5p | DGKE |
| hsa-miR-143-5p | TSHZ2 |
| hsa-miR-143-5p | UNC13A |
| hsa-miR-143-5p | CENPM |
| hsa-miR-143-5p | IBA57 |
| hsa-miR-143-5p | HACD4 |
| hsa-miR-143-5p | ADCY2 |
| hsa-miR-143-5p | SPIB |
| hsa-miR-143-5p | ELK1 |
| hsa-miR-143-5p | TCEANC2 |
| hsa-miR-143-5p | RBM23 |
| hsa-miR-143-5p | NCBP3 |
| hsa-miR-143-5p | HEYL |
| hsa-miR-143-5p | SIK2 |
| hsa-miR-143-5p | NKPD1 |
| hsa-miR-143-5p | PLEKHM3 |
| hsa-miR-143-5p | GNB4 |
| hsa-miR-143-5p | KLF10 |
| hsa-miR-143-5p | RNF216 |
| hsa-miR-143-5p | GPR155 |
| hsa-miR-143-5p | ESCO1 |
| hsa-miR-143-5p | CGN |
| hsa-miR-143-5p | FKBP8 |
| hsa-miR-143-5p | HDAC7 |
| hsa-miR-143-5p | MAP3K2 |
| hsa-miR-143-5p | MAPK8IP3 |
| hsa-miR-143-5p | NPLOC4 |
| hsa-miR-143-5p | AK3 |
| hsa-miR-143-5p | CAPZA1 |
| hsa-miR-143-5p | DCP1A |
| hsa-miR-143-5p | HNRNPUL1 |
| hsa-miR-143-5p | KRI1 |
| hsa-miR-143-5p | MLXIP |
| hsa-miR-143-5p | PIK3R2 |
| hsa-miR-143-5p | ZNF347 |
| hsa-miR-143-5p | TMEM120B |
| hsa-miR-1275 | IGF1R |
| hsa-miR-1275 | IGF1R |
| hsa-miR-1275 | IGF1R |
| hsa-miR-1275 | IGF1R |
| hsa-miR-1275 | ANKRD13B |
| hsa-miR-1275 | ANKRD13B |
| hsa-miR-1275 | ANKRD13B |
| hsa-miR-1275 | MINK1 |
| hsa-miR-1275 | HNRNPAB |
| hsa-miR-1275 | WNT9B |
| hsa-miR-1275 | ILK |
| hsa-miR-1275 | MEN1 |
| hsa-miR-1275 | MEN1 |
| hsa-miR-1275 | SUV39H1 |
| hsa-miR-1275 | DDA1 |
| hsa-miR-1275 | THSD4 |
| hsa-miR-1275 | THSD4 |
| hsa-miR-1275 | MYL12A |
| hsa-miR-1275 | UBE2V1 |
| hsa-miR-1275 | UBE2N |
| hsa-miR-1275 | TMEM189-UBE2V1 |
| hsa-miR-1275 | TMEM189 |
| hsa-miR-1275 | RICTOR |
| hsa-miR-1275 | RAB15 |
| hsa-miR-1275 | PKM |
| hsa-miR-1275 | PKM |
| hsa-miR-1275 | NACC1 |
| hsa-miR-1275 | MEX3A |
| hsa-miR-1275 | LRRC58 |
| hsa-miR-1275 | KMT2D |
| hsa-miR-1275 | KMT2D |
| hsa-miR-1275 | KLHL28 |
| hsa-miR-1275 | KLHL28 |
| hsa-miR-1275 | KLHL28 |
| hsa-miR-1275 | KLHL28 |
| hsa-miR-1275 | C19orf47 |
| hsa-miR-1275 | BRPF1 |
| hsa-miR-1275 | ACTB |
| hsa-miR-1275 | CITED4 |
| hsa-miR-1275 | CITED4 |
| hsa-miR-1275 | CITED4 |
| hsa-miR-1275 | STMN3 |
| hsa-miR-1275 | STMN3 |
| hsa-miR-1275 | STMN3 |
| hsa-miR-1275 | ABCC6 |
| hsa-miR-1275 | ABCC6 |
| hsa-miR-1275 | RTBDN |
| hsa-miR-1275 | STEAP3 |
| hsa-miR-1275 | STEAP3 |
| hsa-miR-1275 | DLGAP3 |
| hsa-miR-1275 | DLGAP3 |
| hsa-miR-1275 | DLGAP3 |
| hsa-miR-1275 | B3GALNT2 |
| hsa-miR-1275 | PRPF4B |
| hsa-miR-1275 | PKNOX2 |
| hsa-miR-1275 | CLIP2 |
| hsa-miR-1275 | PADI1 |
| hsa-miR-1275 | SLC10A7 |
| hsa-miR-1275 | SLC10A7 |
| hsa-miR-1275 | SLC10A7 |
| hsa-miR-1275 | RAB11FIP4 |
| hsa-miR-1275 | PHLDA3 |
| hsa-miR-1275 | PHLDA3 |
| hsa-miR-1275 | DIRAS2 |
| hsa-miR-1275 | DIRAS2 |
| hsa-miR-1275 | ORMDL3 |
| hsa-miR-1275 | ORMDL3 |
| hsa-miR-1275 | VPS37D |
| hsa-miR-1275 | PRELP |
| hsa-miR-1275 | RAP1GAP2 |
| hsa-miR-1275 | RPP25 |
| hsa-miR-1275 | LPCAT3 |
| hsa-miR-1275 | HOXB5 |
| hsa-miR-1275 | P3H2 |
| hsa-miR-1275 | P3H2 |
| hsa-miR-1275 | TBXA2R |
| hsa-miR-1275 | TAOK1 |
| hsa-miR-1275 | SH3PXD2A |
| hsa-miR-1275 | AP2M1 |
| hsa-miR-1275 | ARHGDIA |
| hsa-miR-1275 | ARPP19 |
| hsa-miR-1275 | CASTOR2 |
| hsa-miR-1275 | CPSF7 |
| hsa-miR-1275 | FBRS |
| hsa-miR-1275 | NFIX |
| hsa-miR-1275 | SUSD6 |
| hsa-miR-1275 | SZRD1 |
| hsa-miR-1275 | UBE2Z |
| hsa-miR-1275 | UBL5 |
| hsa-miR-1275 | UBL5 |
| hsa-miR-1275 | UBTF |
| hsa-miR-1275 | TLN1 |
| hsa-miR-126-3p | SPRED1 |
| hsa-miR-126-3p | SPRED1 |
| hsa-miR-126-3p | SPRED1 |
| hsa-miR-126-3p | SPRED1 |
| hsa-miR-126-3p | SPRED1 |
| hsa-miR-126-3p | SPRED1 |
| hsa-miR-126-3p | SLC45A3 |
| hsa-miR-126-3p | RGS3 |
| hsa-miR-126-3p | RGS3 |
| hsa-miR-126-3p | RGS3 |
| hsa-miR-126-3p | CRK |
| hsa-miR-126-3p | CRK |
| hsa-miR-126-3p | CRK |
| hsa-miR-126-3p | CRK |
| hsa-miR-126-3p | CRK |
| hsa-miR-126-3p | CRK |
| hsa-miR-126-3p | CRK |
| hsa-miR-126-3p | PIK3R2 |
| hsa-miR-126-3p | PIK3R2 |
| hsa-miR-126-3p | PIK3R2 |
| hsa-miR-126-3p | PIK3R2 |
| hsa-miR-126-3p | PIK3R2 |
| hsa-miR-126-3p | PIK3R2 |
| hsa-miR-126-3p | IRS1 |
| hsa-miR-126-3p | IRS1 |
| hsa-miR-126-3p | IRS1 |
| hsa-miR-126-3p | IRS1 |
| hsa-miR-126-3p | IRS1 |
| hsa-miR-126-3p | IRS1 |
| hsa-miR-126-3p | IRS1 |
| hsa-miR-126-3p | SLC7A5 |
| hsa-miR-126-3p | SLC7A5 |
| hsa-miR-126-3p | SLC7A5 |
| hsa-miR-126-3p | SLC7A5 |
| hsa-miR-126-3p | ADAM9 |
| hsa-miR-126-3p | ADAM9 |
| hsa-miR-126-3p | ADAM9 |
| hsa-miR-126-3p | ADAM9 |
| hsa-miR-126-3p | ADAM9 |
| hsa-miR-126-3p | ADAM9 |
| hsa-miR-126-3p | CXCL12 |
| hsa-miR-126-3p | RBMX |
| hsa-miR-126-3p | NFKBIA |
| hsa-miR-126-3p | AKT2 |
| hsa-miR-126-3p | SZRD1 |
| hsa-miR-126-3p | PLAGL2 |
