# Supplementary figures and images for: Development and clinical validation of a 3-miRNA signature to predict prognosis of gastric cancer
Source: PeerJ. 2021 Feb 3;9:e10462. doi: 10.7717/peerj.10462 (PMC7866890; doi:10.7717/peerj.10462)

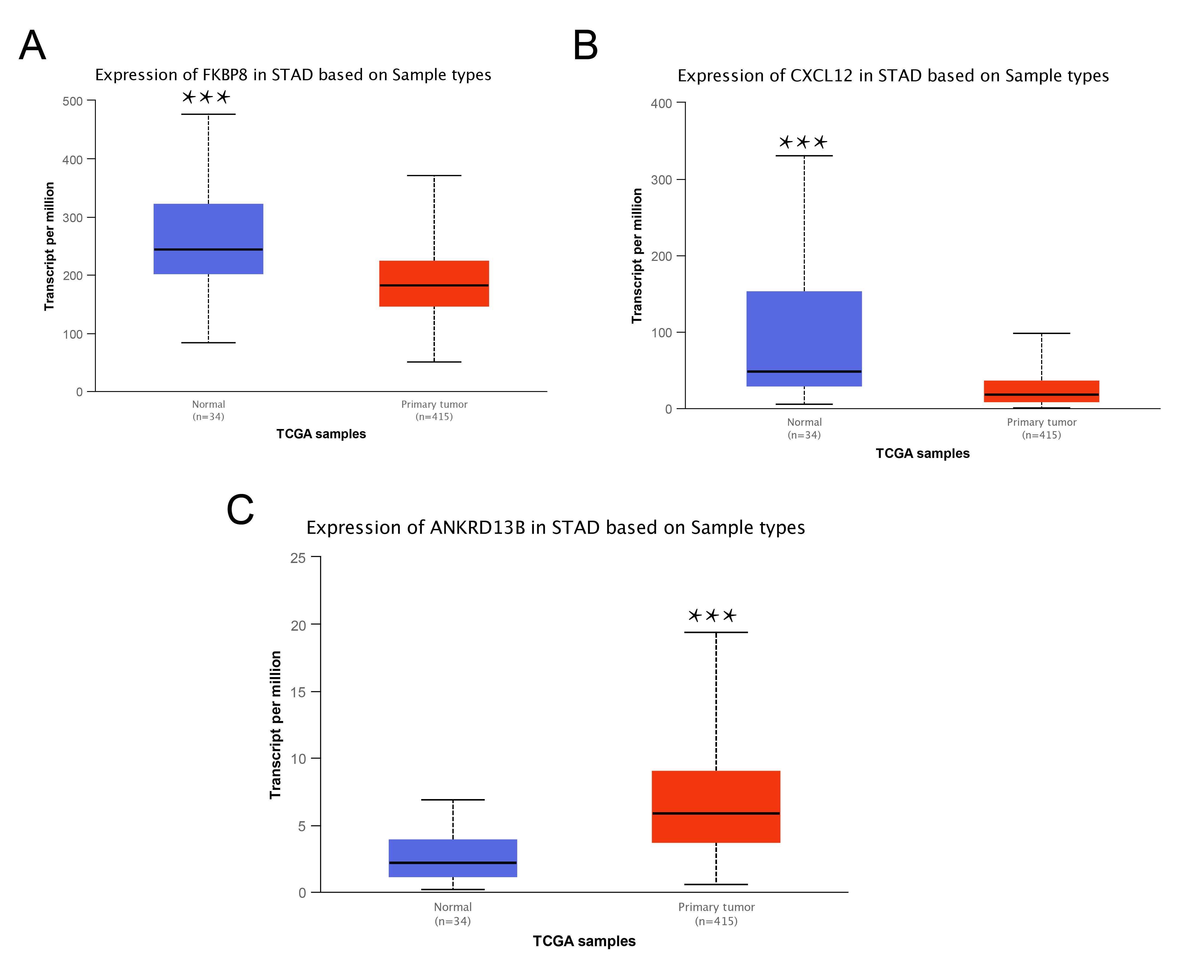

Supplement: Figure S1 [file peerj-09-10462-s004.png]
